# Extract from *Eucommia ulmoides* Oliv. Leaves alleviates APAP-induced acute liver injury via reshaping gut microbiota and attenuating microbial bile acids metabolism disorder

**DOI:** 10.1186/s13020-026-01496-9

**Published:** 2026-10-14

**Authors:** Dan He, Hai-chao Zhang, Bing-bing Shen, Di Zhao, Xue-yang Tang, Jing Xie, Rong-rong Zhou, Hong-liang Zeng, Shui-han Zhang

**Affiliations:** 1 Hunan Academy of Chinese Medicine, Hunan University of Chinese Medicine, Changsha, Hunan China; 2 Department of Pharmacy, Women’s Hospital School of Medicine Zhejiang University, Hangzhou, China; 3 Institute of Chinese Medicine Resources, Hunan Academy of Chinese Medicine, Changsha, Hunan China; 4 The Affiliated Hospital of Hunan Academy of Traditional Chinese Medicine, Changsha, China; 5 Center of Medical Laboratory Animal, Hunan Academy of Chinese Medicine, Changsha, Hunan China

**Keywords:** *Eucommia ulmoides* Oliv. Leaves, Acetaminophen, Acute liver injury, Gut microbiota, Bile acids-targeted metabolism, Farnesoid X Receptor

## Abstract

**Background:**

Increasing studies suggest that supplementing flavonoids from *Eucommia ulmoides* Oliv. Leaves (EUL) exerts liver protective effects via reshaping gut microbiota and regulating bile acids (BAs) metabolism. However, the relationship between EUL, gut microbiota and BAs metabolism remains unclear and the potential mechanisms of action has not been expounded clearly.

**Purpose:**

To demonstrate the important role of gut microbiota and microbial BAs metabolism in alleviation acetaminophen-induced liver injury (AILI) by EUL.

**Methods:**

AILI mice model was employed to evaluate the therapeutic efficacy of EUL. The hepatic function was assessed, and biological samples were collected for histopathological examination, bacterial 16S rRNA sequencing analysis, BAs-targeted metabolomics, and the evaluation of the intestinal barrier function. Additionally, antibiotic cocktail-triggered gut microbiota deficiency and fecal microbiome transplantation were investigated to the relationship of molecular mechanistic between the protective efficiency of EUL in AILI, gut microbiota, and the metabolism of microbial BAs.

**Results:**

EUL alleviate liver damage, involving reduction of liver function index, down-regulation of inflammatory cytokines and oxidative stress index, and restoration of pathological changes of liver tissue. EUL also improved intestinal barrier dysfunction and reshaped gut microflora dysbiosis in AILI mice. Moreover, antibiotic cocktail-triggered intestinal microbiota deficiency and fecal microbiome transplantation (FMT) evidenced that the liver protection of EUL was tightly related with gut microbiota. BAs-targeted metabolomics assay demonstrated that EUL mitigated the altered metabolism of microbial BAs during the development of AILI. EUL promoted the abundance of *Firmicutes* phylum, and elevating the beneficial microbes in *Ruminococcaceae* and *Lachnospiraceae* family, which were associated with up-regulated proportion of secondary bile acids (including DCA, NorDCA and 7-ketoLCA), leading to the reduction intestinal inflammation and preservation intestinal integrity. In particular, EUL enriched the beneficial microbes in *Lachnoclostridium, Lachnospiraceae_UCG-006, [Eubacterium]_xylanophilum_group, Oscillibacter, Ruminiclostridium,* and *Desulfovibrio* genera. Furthermore, EUL administration increased levels of CDCA, which could activate FXR-FGF15, enabling to decreased synthesis of BAs in the liver and promoting excretion.

**Conclusions:**

EUL supplementation alleviate AILI by reshaping the dysfunctional gut microbiota and attenuating disordered BAs metabolism, resulting to improvements in BAs enterohepatic circulation and gut barrier function. The mechanism may be closely related to the gut microbiome-BAs-FXR signaling in gut-liver axis.

**Supplementary Information:**

The online version contains supplementary material available at https://doi.org/10.1186/s13020-026-01496-9.

## Introduction

Acute liver failure (ALF) is a rapidly progressive liver disease that often results in multi-organ failure and even death. It has been reported that acetaminophen-induced liver injury (AILI) accounts for 46% of all ALF patients in the United States, which is more than four times the incidence of liver injury caused by other specific drugs [47]. At therapeutic dose (1–4 g/d), acetaminophen (APAP) is commonly used in clinical practice as an antipyretic and analgesic and is mainly metabolized through sulfation and glucuronidation. However, in cases of long-term use or unintentional overdose, APAP is predominantly metabolized by cytochrome P450 (CYP) enzymes in the liver into the cytotoxic metabolite N-acetyl-p-benzoquinone imine (NAPQI), which depletes hepatic glutathione (GSH), induces oxidative stress, promotes the production of pro-inflammatory cytokines (such as TNF-α, IL-6, and IL-1β), and ultimately leads to hepatocyte death [45, 65, 93].

More than 100 trillion bacteria colonize the human intestine, where they interact with the liver through metabolic, immune, and neuroendocrine crosstalk, thereby forming the gut-liver axis [43]. Tight junctions (TJs) in the intestinal epithelium constitute a natural barrier of the intestine [57]. In mice, APAP intoxication significantly decreased the protein levels of the tight junction proteins occludin and claudin-4 and triggered apoptosis of intestinal crypt stem cells [59, 67]. Damage to the intestinal barrier leads to increased bacterial translocation and impaired bile acid metabolism, which may facilitate the delivery of inflammatory cytokines and chemokines to the liver [57]. Previous studies have also shown that gut microbiota dysbiosis increases individual susceptibility to AILI [67]. Specifically, at the phylum level, the *Firmicutes*/*Bacteroidetes* ratio (F/B) was obviously declined in AILI mice [24]. Supplementation with *Lactobacillus* or *Akkermansia muciniphila* has been shown to alleviate AILI by alleviating hepatic oxidative stress and inflammation [54, 84]. Therefore, maintaining gut microbiota homeostasis and restoring intestinal barrier integrity may be important strategies for the prevention and treatment of liver injury.

Bile acids (BAs) are synthesized in liver cells and secreted into intestinal tract as conjugated BAs, and then transformed into secondary BAs by intestinal bacteria [29, 69]. Ninety-five percent of bile acids are reabsorbed in the ileum and transported back to the liver through the enterohepatic circulation, forming the liver-gut" axis [69]. As a multifunctional biomolecule, BAs contribute to energy homeostasis, activate nuclear receptors, and regulate inflammation in the liver and intestine [36, 68, 77]. Recently, BAs has been demonstrated to play a vital role in AILI recovery [7, 34]. APAP is mainly metabolized by liver and absorbed by intestine [56]. Once liver injury occurs, BAs metabolism disorder breaks the balance between primary BAs and secondary BAs in the liver and intestine, and the proportion of intestinal bacteria changes [48]. Therefore, the intestine is exposed to high concentrations of APAP earlier than the liver, resulting in impaired intestinal barrier and dysbacteriosis, and the changed microbial composition will exacerbate the hepatotoxicity of APAP [67]. The lower ratio of secondary to primary BAs allow neutrophils to gather in the liver, causing inflammatory cell infiltration and leading to hepatocyte death [22, 99]. Altogether, shaping a healthy intestinal microbiota, maintaining a positive "liver-gut" metabolic order of BAs, and enhancing the ability to resist oxidative damage may provide adaptive protection for liver damage caused by APAP, which is expected to become an effective strategy to prevent and alleviate AILI. Farnesoid X receptor (FXR) is the dominant regulator of BAs biosynthesis and "liver-gut" circulation [44]. Under normal physiological conditions, hepatic FXR regulates the level of BAs in the liver and intestine, and limits the accumulation of BAs in the liver [91]. The ileum FXR couples BAs, such as chenodeoxycholic acid (CDCA), to be responsible for the excretion and reabsorption of BAs in the intestine, limiting the concentration of BAs in cells [10]. Activating intestinal FXR could up-regulate the expression of fibroblast growth factor-19/15 (FGF-19 in human, FGF-15 in mice), thereby inhibiting the expression of CYP7A1 in liver and the synthesis of BAS in liver cells [3, 37]. The orderly transmission of "FXR-FGF19/15" signals in the hepatointestinal axis benefits from the shaping of a healthy intestinal environment. For example, *Lactobacilli* and *Firmicutes*, have been shown to adapt to the metabolic changes of BAs in the liver, thereby activating the feedback signal of FXR-FGF in the intestine [63]. Moreover, the activation of FXR is participated in the expression of antioxidant genes to up-regulate the level of GSH, contributing to reduce NAPQI-induced hepatotoxicity [52]. Therefore, the restoration of "FXR-FGF19/15" signal by correcting the intestinal microbiota is a potential and effective approach to reconstruct the benign liver-intestinal circulation of BAs.

According to the “Compendium of Materia Medica”, *Eucommia ulmoides* Oliv. is an essential medicine for tonifying the liver and kidney, with the function of protecting and nourishing the liver [61, 75]. *Eucommia ulmoides* Oliv. Leaves are similar to *Eucommia ulmoides* Oliv which commonly used as functional food or medicine in China [79]. *Eucommia ulmoides* Oliv. Leaves extraction is rich in flavonoids, phenols, triterpenes, and polysaccharides that displayed beneficial effects on hypertension, nonalcoholic fatty liver disease, atherosclerosis and rheumatoid arthritis [23, 27, 30, 86]. In particular, flavonoids from *Eucommia ulmoides* Oliv. Leaves (EUL), such as quercetin 3-O-morin, rutin and isoquercetin, show the effects of inhibiting inflammation, oxidative stress and transaminase elevation [11, 81, 98]. Recent studies shared that EUL exhibited effective effects in non-alcoholic fatty liver disease and CCl4-caused hepatic damage [23, 35, 46]. Increasing study suggest that the protective factors for EUL are partially attributed to the optimization of intestinal bacterial community structure and the maintenance of intestinal homeostasis [10, 97, 101]. In addition, EUL could alleviate gut inflammation by regulating the composition of BAs, which promoted the expression of tight junction proteins, reducing intestinal barrier disruption [96]. Taken together, EUL possessed anti-inflammation, anti-oxidation, hepatoprotective as well as potential probiotic effects. However, it is unclear whether flavonoids from EUL could alleviate AILI that is related to intestinal microbiota and BAs metabolism.

Therefore, we supposed that supplement with a flavonoids-enriched EUL could alleviate the AILI by reshaping gut microbial composition and restoring microbial BAs homeostasis. To confirm this conjecture, APAP-induced acute liver injury in mice was established, and the liver function indexes, inflammatory cytokines and oxidative stress indexes in both liver and serum, as well as pathological changes of liver tissue were systematically observed. 16S rRNA amplicon sequencing and BAs-targeted metabolomics technology were employed to respectively investigate the contributions of EUL on gut microbiota and microbial BAs. In addition, antibiotic cocktail-triggered depletion of the gut microflora and fecal microbiome transplantation (FMT) were also performed to explore the relationship between intestinal microecology and the hepatoprotective effects of EUL on alleviating AILI.

## Materials and methods

### Preparation of *Eucommia ulmoides* Oliv. Leaves Extract

*Eucommia ulmoides* Oliv. Leaves was collected from Hunan (Lot. 20210506) and authenticated by Prof. Hao-Liu from the Hunan Academy of Chinese Medicine. In this study, EUL was extracted and purified according to the following optimization steps (Fig. S2A). In short, 500 g dried *Eucommia ulmoides* Oliv. Leaves was soaked in 1500 mL water for 30 min, and extracted for 2 h at 100 ℃ to obtain Extract 1. The residues were further extracted with 1200 mL water at 100 ℃ for 1.5 h to obtain Extract 2. Then, extract 1 and 2 were mixed thoroughly and freeze-dried to produce water decoction. The crude water extract was purified by a column (75 mm × 450 mm) packed with AB-8 macroporous adsorption resin. The macroporous resin column was first soaked in absolute ethanol overnight and then eluted with distilled water. After the crude extract was injected, the column was then eluted successively with water, aqueous ethanol (30%, 50%, 70%, 90%, 100%) at a flow rate of 3.0–4.0 mL/min. Each elution part was collected and concentrated by rotary evaporation at 45 °C and then lyophilized to dryness. The yield rate of the 50% extract was 4.9%, i.e. 500 g raw materials yielded 24.6 g EUL. Detailed compositional characterization of EUL is presented in Tab. S1 and Fig. S1.

### Animals and drug administration

C57BL/6J male mice aged 6–8 weeks old (weight 18–20 g) with specific pathogen free (unless otherwise stated) were obtained from the Hunan SJA Laboratory Animal Co. Ltd., (Changsha, China, SYXK < Xiang > 2020-0008). Animal study was performed to follow the Guidelines for Animal Experimentation of Hunan Academy of Chinese Medicine (approval number: 20210064).

After 7d of adaptive feeding, experimental animals were randomly divided into 6 groups: (1) healthy control group (Ctrl, n = 14); (2) acetaminophen-treated group (400 mg/kg/d, APAP, n = 14; M3301-02, AbMole Bioscience, USA); (3) high-dose EUL (100 mg/kg, APAP + EULH, n = 14); (4) medium-dose EUL (50 mg/kg, APAP + EULM, n = 7); (5) low-dose EUL (25 mg/kg, APAP + EULL n = 7); (6) positive control group (50 mg/kg, NAC, n = 7; M5385-05, AbMole Bioscience, USA). The Ctrl group and APAP group mice were gavaged orally with 0.2 mL distilled water daily, while the drug group was given equal volume of EULH, EULM, EULL or NAC. The intervention lasted 14 days. On the 15th day, according to previous studies, the acute liver injury model induced by APAP was established [24]. In brief, after fasting 12 h, animal in the groups APAP, APAP + EULH, APAP + EULM, APAP + EULL and APAP + NAC were administrated (i.p) with 400 mg/kg APAP (dissolved in normal saline), while those in Ctrl were given an equivalent volume of normal saline. Fasting was continued and animals were euthanized 12 h after the injection of APAP. The liver was dissected and weighed to calculate the liver indices. Liver index (mg/g) = liver mass (mg)/animal body-weight (g). The blood, liver tissue, ileal tissue and cecal contents were then collected and stored in liquid nitrogen for further analysis. Then serum samples were obtained by centrifuging the coagulated blood at 4 °C, 3000 r/min, continued 10 min.

### Fecal microbiota transplantation and antibiotic treatment

Fecal transplant was conducted as previously described [15]. The recipient mice (APAP + FEUL group, n = 7) were fed daily with fresh transplant fecal from donor mice administrated with 100 mg/kg EUL. In order to obtain stable intestinal bacterial components, fresh graft materials were prepared within 10 min before oral gavage. Briefly, the fresh fecal samples collected from donor mice were dissolved in sterile normal saline (200 mg/mL), and homogenized 1 min by vortex. The mixture was then centrifuged at 500 rpm for 3 min to remove particles for administration. APAP + FEUL group (n = 7) and APAP + ABX + FEUL (n = 7) group were gavaged with fecal transplant material daily during the 14d experiment. ﻿The APAP and EUL groups used in the FMT-related experiments were derived from the same animal cohort presented in the initial pharmacodynamic evaluation.

As previously described [49], antibiotic cocktail (ABX) was prepared by metronidazole (100 mg/kg), vancomycin (50 mg/kg), Neomycin (100 mg/kg), ampicillin (100 mg/kg), which were dissolved in 0.5% CMC-Na. APAP + ABX group (n = 7), APAP + ABX + EUL(n = 7) group and APAP + ABX + FEUL group were received daily oral gavage of ABX for 2 weeks before the experiment to deplete intestinal bacteria. During this period, fresh ABX solution was prepared daily.

### Biochemical measurements

Aspartate aminotransferase (AST, Lot. F2ETP9A12A) and alanine aminotransferase (ALT, GEY611VCK2) were purchased from Elabscience Biotechnology Co.,Ltd (Wuhan, China) to assay liver injury. The concentrations of serum glutathione peroxidase (GSH-Px, Lot. 20200918) and malondialdehyde (MDA, Lot. 20200704) were measured using assay kits (ZCIBIO Technology Co., Ltd., Shanghai, China).

### Bio-plex assay

The concentrations of FGF15 (Lot. 202205A06) in serum and liver were detected using ELISA kits (ZCIBIO Technology Co., Ltd., Shanghai, China). The serum and liver cytokine profiles (including TNF-α, IL-6, IL-1β, CXCL10, CCL2, CCL3, CCL4) were monitored through Mouse ProcartaPlex Mix&Match 7-plex (Lot. 304,878–000, Thermo Fisher Scientific).

### Histological analysis

In order to observe the histopathological characteristics of the liver, routine H&E staining was applied after deparaffinization [103]. In brief, after being fixed in 4% paraformaldehyde for more than 48 h, samples of liver tissues were embedded in paraffin, and then sliced in 4 µm thickness. A light microscope was employed to record the images (Nikon Eclipse Ci-L, 200× magnification).

### Immunohistochemistry and immunofluorescence

The expression of 3-Nitrotyrosin, and CK-19 in liver tissue and the claudin-1, occluding, ZO-1 and IBABP in ileum were detected by immunohistochemistry. The paraffin embedded sample sections were dewaxed and then dipped into EDTA antigen repair solution (pH 8.0) to thermally repair the antigen, followed by addition of 3% H_2_O_2_ to inactivate the endogenous enzyme. After blocking with 5% sheep serum, primary antibodies against 3-Nitrotyrosinr (BIOSS, Bs-8551R, 1:3000), CK-19 (Proteintech, 10,712–1-AP, 1:200), Claudin-1 (Affinity, AF01878, 1:100), Occluding (Servicebio, GB111401, 1:1000), ZO-1 (Affinity, AF09017, 1:200) and IBABP (Servicebio, GB113145, 1:1000) and goat anti-mouse secondary antibodies were used, respectively. Then DAB (Servicebio, G1212) and hematoxylin were sequentially employed to stain the sample. A DM2000 LED microscope and Image J software (ver.6.0) were used to record the stain and calculate the DAB positive stain respectively.

Immunofluorescence was performed as previous study from [103]. After deparaffinization, liver tissue sections were incubated with anti-mouse F4/80 (Servicebio, gb113373, 1:1000) primary antibody for overnight, while ileum tissue sections were treated with anti-mouse FGF15 (Santa, Sc-398338, 1:200). Then liver and ileum tissue sections were conjugated with Cy3 goat anti-rabbit IgG antibody (Beyotime, A0516) and FITC goat anti-mouse antibody (Servicebio, GB22301) respectively for 1 h. Afterwards, DAPI was added for nuclear staining. Fluorescence microscope was used for observation.

### Real-time qPCR

The total RNA of liver or ileum tissues was isolated by Trizol reagent (TIANGEN). Nano-200 spectrophotometer (ALLSHENG, Hangzhou, China) was employed to measure the RNA concentration. cDNA templates were set up using reverse transcription of total RNA samples. Supplementary Table 2 shows the forward and reverse sequences of qPCR primers (Tsingke Biotechnology Co., Ltd., Beijing, China). The quantitative real-time PCR reaction mixture was established by PerfectStart Green qPCR SuperMix (TransGen Biotech, Beijing, China). The PCR amplification was carried out in an ABI Real-Time PCR System (Thermo Fisher Scientific, USA). GAPDH is applied to normalize the target gene value. The relative expression level of target genes was displayed as fold changes relative to Ctrl group.

### 16S rRNA analysis

At the end of the experiment, cecal content samples were obtained from each animal and stored at − 80 °C for further analysis. Sample DNA was extracted according to the E.Z.N.A. Soil DNA Kit (D5625-02, Omega, Inc., USA). The Thermo NanoDrop 2000 micro spectrophotometer was used to detect the sample concentration. In order to estimate the microbiota composition, PCR was used to amplify the V3-V4 region of microbial 16S rRNA gene, and the primer was 338F-806R (Forward 5′-ACTCCTACGGGAGGCAGCAG-3′ and Reverse 5′-GGACTACHVGGGTWTCTAAT-3′). After amplification, the PCR products were purified via Vazyme VAHTSTM DNA Clean Beads and quantified by Microplate reader (BioTek, FLx800). TruSeq Nano DNA LT Library Prep Kit from Illumina Company was used to prepare the sequencing library. Then samples were inspected with Agilent High Sensitivity DNA Kit.

### Targeted bile acid quantification

Lyophilized cecal content samples were homogenized with 400 μL methanol (− 20 ℃) at 55 Hz for 2 min using a High-throughput tissue grinder (MEBI INSTRUMENTS, Zhejiang, China). The samples were cryogenically sonicated for 30 min and centrifuged at 12,000 rpm for 10 min at 4 °C (supernatant 1). Take 300 μL supernatant 1 added 600 μL water mixed and vortex oscillated for 30 s. Take the above 300 μL mixture and filter it through 0.22 membrane, vortex it for 30 s, centrifuge it at 12,000 rpm at 4 ℃ for 10 min (supernatant 2). After centrifugation, 160 μL of 30% methanol solution was added to 40 μL of the supernatant 2. A total of 5 μL sample were subsequently taken to record a bile acid profile.

### Statistical analysis

The experimental data were analyzed via SPSS 21.0 software. Student’s T-test was used for the statistical analysis between two groups, while one-way analysis of variance (ANOVA) was applied for more than two groups. In brief, data were evaluated for conformity to a normal distribution. If they met the normal distribution, they were evaluated for variance, otherwise they were accessed for Kruskal–Wallis test. The LSD test was conducted on the assumption of equal variances, otherwise, the Games-Howell test was applied to the data with unequal variances. Statistical significance shall be established at *P* < 0.05 or *P* < 0.01. Normally distributed data are shown by mean ± SEM, while skewed data are displayed as median (min–max). Different assays were performed using different subsets of animals from the same experimental cohort; therefore, the value of n may vary across figures. The actual sample size used for each analysis has been clearly indicated in the corresponding figure legend.

## Results

### Quantitative and qualitative analysis of the major components in EUL

**Fig. 1:**
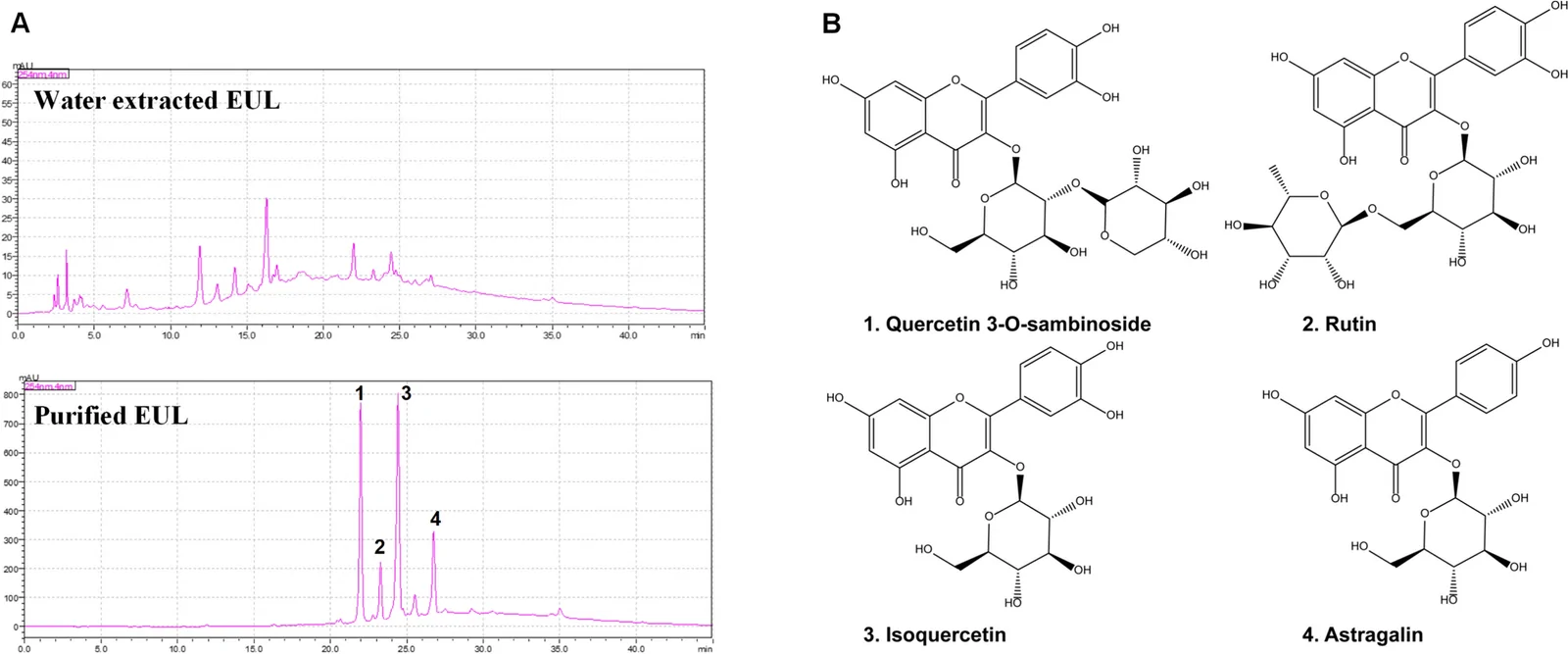
Chemical composition analysis and identification of EUL. EUL, flavonoids from *Eucommia ulmoides* Oliv. Leaves. **A** Typical high-performance liquid chromatography (HPLC) chromatograms of water extracted EUL and purified EUL. mAU: milli-absorbance unit. **B** Chemical structures of the four major flavonoids in EUL

In this study, we optimized the extraction and purification process of EUL, and obtained a flavonoids-enriched extract with quercetin 3-O-sambinoside, rutin, isoquercetin and astragalin (Fig. 1). The content of each compound is listed in Table 1.

**Table 1:** Content date of EUL by HPLC

Name	Calibration curve	Range (μg/mL)	Coefficient (R^2^)	Concentration (μg /mL)
Quercetin 3-O-sambinoside	y = 1 × 10^7^x + 311,436	193.6–1000	0.9995	468
Rutin	y = 8 × 10^6^x + 3565	101–253	0.9999	219
Isoquercretin	y = 1 × 10^7^x + 394,859	56.3~1420	0.9996	508
Astragalin	y = 8 × 10^7^x + 9719.7	42.4–102.2	0.9999	28

### EUL shows hepatoprotective effect on AILI mice

**Fig. 2:**
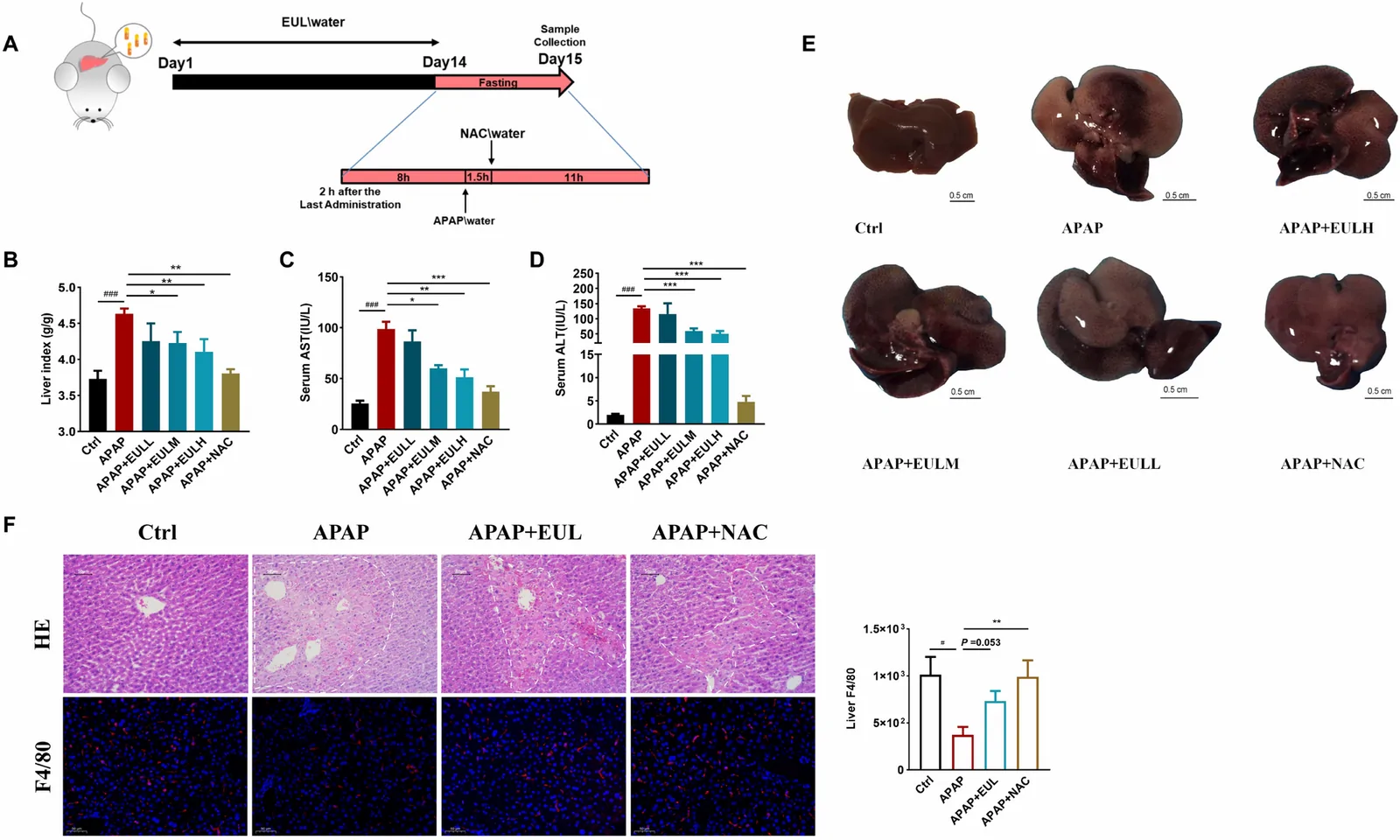
EUL showed hepatoprotective effect on AILI mice. EUL, flavonoids from *Eucommia ulmoides* Oliv. Leaves; AILI, acetaminophen-induced acute liver injury. **A** Animal experimental design. **B**–**D** Liver index; Serum AST; and Serum ALT (n = 7). **E** Representative gross liver appearance. **F** Representative images of hematoxylin and eosin (H&E), and F4/80 immunostaining in liver tissue; scale bar = 50 µm (n = 5); All analyses of were based on a high dose of EUL (100 mg/kg). Data are expressed as the mean ± SEM by ANOVA. ^#^*P* < 0.05, ^###^*P* < 0.001 compared with Ctrl group; ^*^P < 0.05, ^**^P < 0.01, ^***^P < 0.001 compared with APAP group

To investigate the hepatoprotective effect of EUL, APAP-induced acute liver injury (AILI) mice was established, as shown in Fig. 2A. Compared with the Ctrl group, APAP group showed significant elevation in liver index (*P* < 0.001), serum ALT (*P* < 0.001) and AST (*P* < 0.001) activities (Fig. 2B–D). After administered EUL, liver index (*P* < 0.01 or *P* < 0.05), serum ALT (*P* < 0.001) and AST (*P* < 0.01 or *P* < 0.05) activities were decreased a dose-dependent manner.

Additionally, we observed APAP-treated mice liver with uneven surface color and reduced elasticity and smoothness (Fig. 2). Severe 3-nitrotyrosine immunoreactivity was associated with APAP-triggered necrosis [39]. APAP group developed higher levels of 3-nitrotyrosine (*P* < 0.05) (Fig. S3). Those histopathological changes are responses to previous animal models [31, 51]. HE evaluation displayed that EUL pretreatment improved APAP-triggered serious liver including hepatic architecture damage, hepatocyte necrosis and irregularly arrangement (Fig. 2E). As compared to mice in the Ctrl group, APAP treatment caused the numbers of hepatic F4/80-positive cells (*P* < 0.05) significantly declined, while treatment with EUL stimulated the reestablishment of positive cells (*P* = 0.053) in the liver compared with the APAP group (Fig. 2F). This data indicates that EUL involved in attenuating APAP-induced loss of Kupffer cells.

### EUL alleviates APAP-induced oxidative stress and inflammatory response

**Fig. 3:**
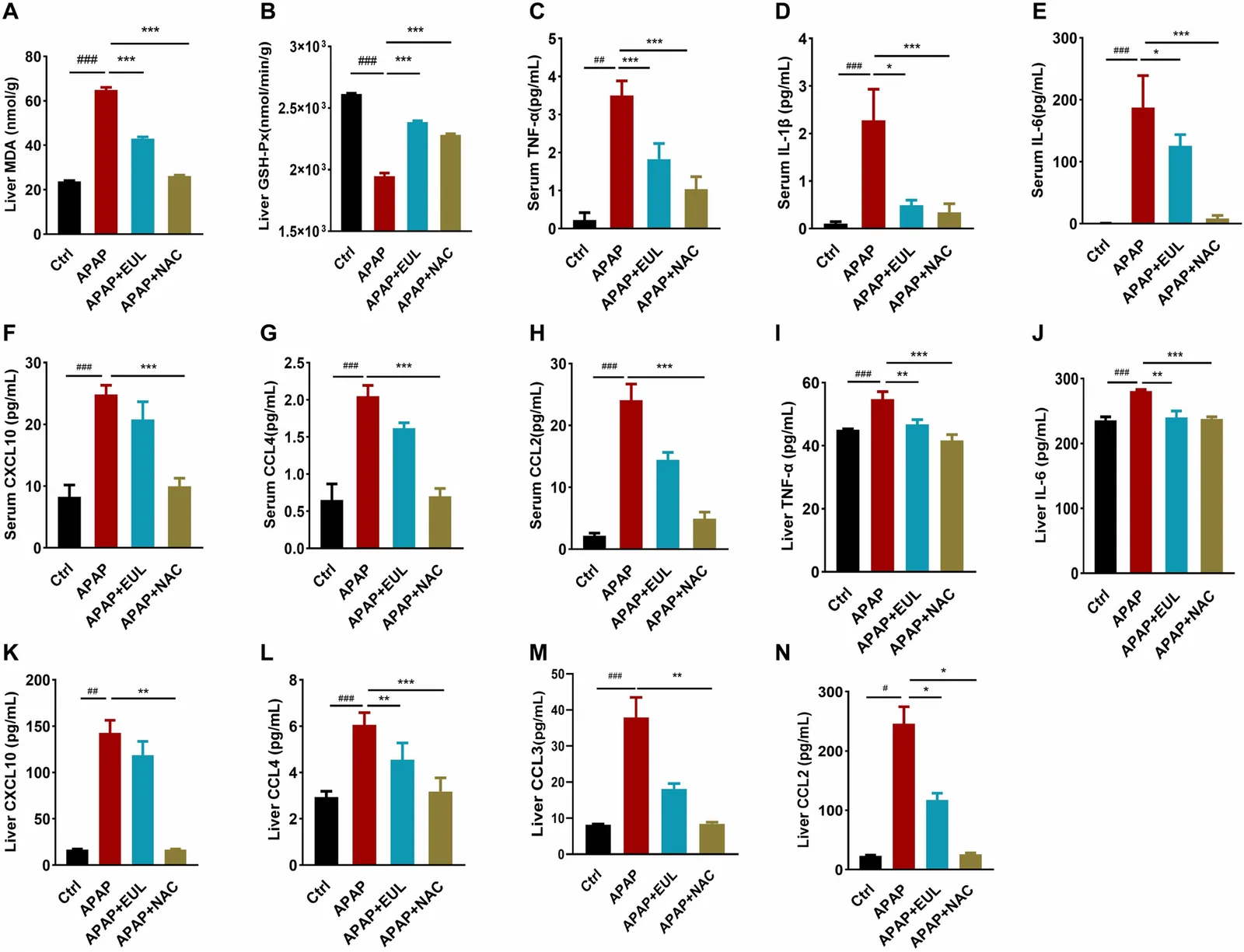
EUL alleviated oxidative stress and the inflammatory in AILI mice. EUL: flavonoids from *Eucommia ulmoides* Oliv. Leaves; AILI: acetaminophen-induced acute liver injury. **A** Liver MDA. **B** Liver GSH-Px. **C** Serum TNF-α. **D** Serum IL-1β. **E** Serum IL-6. **F** Serum CXCL10. **G** Serum CCL4. **H** Serum CCL2. **I** Liver TNF-α. **J** Liver IL-6. **K** Liver CXCL10. **L** Liver CCL4. **M** Liver CCL3. **N** Liver CCL2 (n = 5). All analyses of were based on a high dose of EUL (100 mg/kg). Data are expressed as the mean ± SEM by ANOVA. ^#^*P* < 0.05, ^##^*P* < 0.01, ^###^*P* < 0.001 compared with Ctrl group; ^*^P < 0.05, ^**^P < 0.01, ^***^P < 0.001 compared with APAP group

Oxidative stress and inflammation take important roles in APAP-induced hepatotoxicity [89]. As shown in Fig. 3A, the MDA (*P* < 0.001) content of liver tissue in APAP group was markedly increased as compared with that in the Ctrl group. Additionally, APAP treated mice obviously reduced the activities of GSH-Px (*P* < 0.001) (Fig. 3B). In contrast, EUL treatment restored hepatic antioxidant capacity, including an apparent decrease in liver MDA levels (*P* < 0.001) and a significant increase in liver GSH-Px levels (*P* < 0.001) as compared to APAP group.

The results of the proinflammatory cytokines assay exhibited that serum TNF-α (*P* < 0.01), IL-1β (*P* < 0.001), IL-6 (*P* < 0.001) levels and liver TNF-α (*P* < 0.001), IL-6 (*P* < 0.001) levels in the APAP group were remarkedly up-regulated compared with the Ctrl group (Fig. 3C–N). Similarly, the results of the chemokines estimate shown that serum CXCL10 (*P* < 0.001), CCL4 (*P* < 0.001), CCL2 (*P* < 0.001) levels and liver CXCL10 (*P* < 0.01), CCL4 (*P* < 0.001), CCL3 (*P* < 0.001), CCL2 (*P* < 0.05) levels in the APAP group were distinctly increased compared with the Ctrl group (Fig. 3C–N). It indicated that APAP developed an inflammatory response in the liver. Pretreatment with EUL significantly decreased the serum levels of TNF-α (*P* < 0.001), IL-1β (*P* < 0.05), IL-6 (*P* < 0.05) and down-regulated the liver levels of TNF-α (*P* < 0.01), IL-6 (*P* < 0.01), CCL4 (*P* < 0.05), CCL2 (*P* < 0.05), as compared with APAP group. In addition, APAP + EUL group had a trend to reduce the levels of serum CXCL10, CCL4, CCL2, and declined the liver levels of CXCL10, CCL3, but there was no significant difference. Above results implied that EUL may alleviated APAP-induced hepatotoxicity by reducing oxidative stress injury and decreasing inflammatory response in the liver.

### EUL alleviates APAP-induced ileum injury

Previous studies have shown that the intact intestinal mucosa against the migration of viruses, bacteria, and parasites to extra-intestinal tissues and organs [54, 67]. Immunohistochemistry was employed to detect the expression of tight junction associated proteins (including Claudin-1, Occluding and ZO-1) in ileum (Fig.S4). In contrast to Ctrl group, the low expression of claudin-1 (*P* < 0.05), occludin (*P* < 0.05) and ZO-1 (*P* < 0.01) in ileum were observed in the APAP group, which suggested that the intestinal mucosa integrity was impaired. The APAP + EUL group markedly up-regulated the protein levels of ZO-1 (*P* < 0.01) and occludin *(P* < 0.05), and alleviated the injury to tight junction proteins caused by APAP. Treatment with EUL had a tendency to increase claudin-1 with no significance. Histological and immunohistochemical observations in the ileum suggested that EUL may partially alleviate intestinal barrier impairment.

### EUL modulates microbiota dysbiosis in APAP-induced acute liver injury mice

**Fig. 4:**
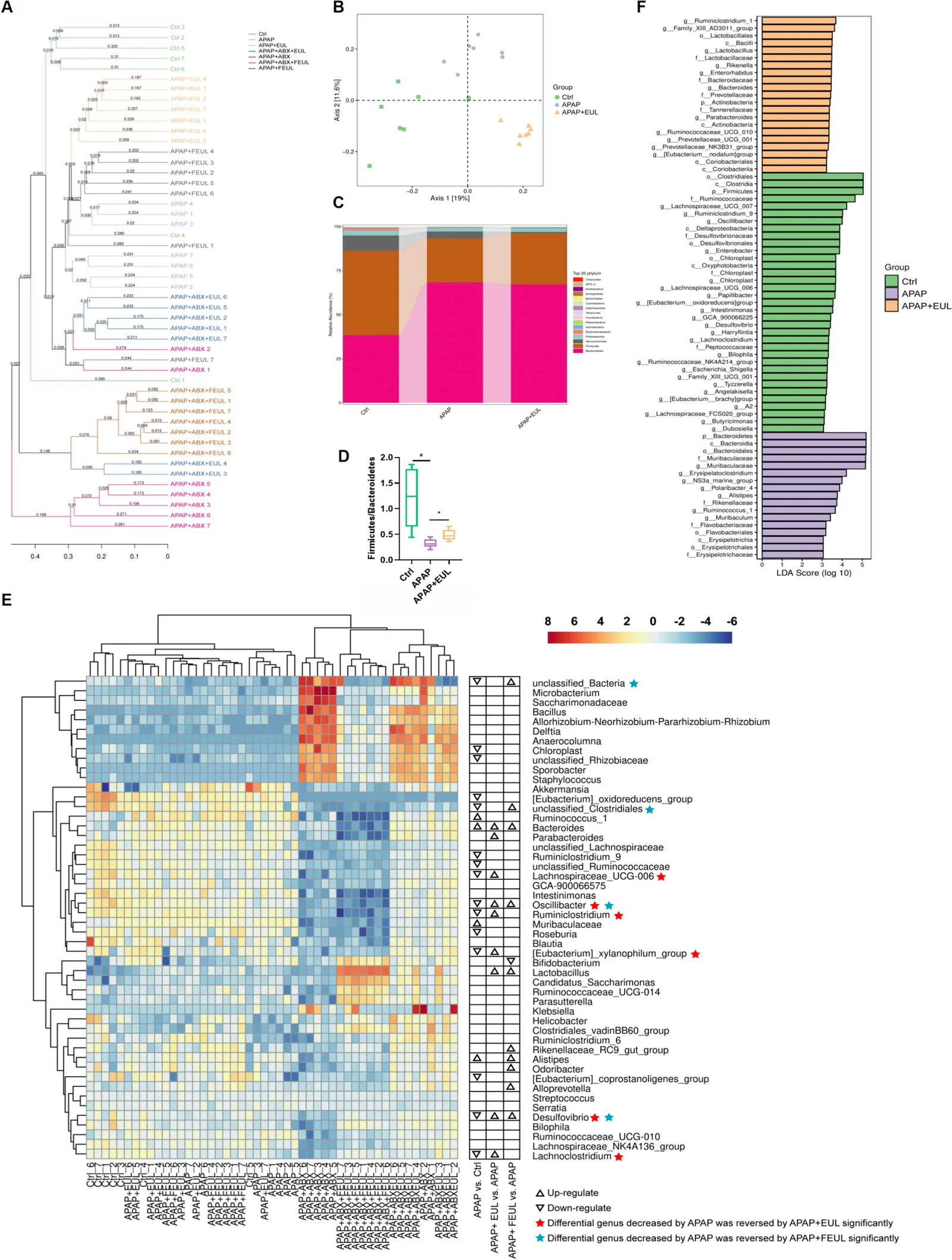
EUL reshape the gut microbiota of AILI mice. EUL: flavonoids from *Eucommia ulmoides* Oliv. Leaves; AILI: acetaminophen-induced acute liver injury. **A** Clustering tree. **B** PCoA plot based on β-diversity analysis. **C** Bacterial taxonomic profiling at the phylum level. **D** *Firmicutes*-to-*Bacteroidetes* (F/B) ratio. **E** Heatmap of relative abundance of top 50 genus. **F** LEfSe analysis. All analyses of were based on a high dose of EUL (100 mg/kg). Data are expressed as the median (min–max) (n = 7). ^#^*P* < 0.05 compared with Ctrl group; ^*^P < 0.05 compared with APAP group by student t test

Intestinal barrier damage is often accompanied by changes in gut microbial composition [95]. In order to investigate whether the reduction of liver injury by EUL is associated with the modulation of intestinal microbiota, we measured the next generation sequence based on 16S rRNA v3-v4 region of cecal content sample. In present work, we obtained 44807 bacterial taxa. The PCoA plot of β-diversity charactered distinct clustering of intestinal microbiota composition between APAP, Ctrl, and APAP + EUL groups (Fig. 4B). Furthermore, clustering tree based on the Bray–Curtis similarity matrix displayed that the distance among Ctrl group and EUL group was relatively closer (Fig. 4A). The relative abundance of gut microbiota in different level was analyzed. At the phylum level, most abundant phyla were *Bacteroidetes, Firmicutes* and *Verrucomicrobia* (Fig. 4C). Consistent with previous studies, APAP-treated mice developed higher abundances of *Bacteroidetes* (*P* < 0.001) and shown a remarkable decline in relative abundance of *Firmicutes* (*P* < 0.05), *Cyanobacteria* (*P* < 0.05) and *Firmicutes-to-Bacteroidetes* (F/B) ratio (*P* < 0.05) (Fig. 4D) as compared to Ctrl group, whereas treatment with EUL considerably increased *Firmicutes* (*P* < 0.05), *Cyanobacteria* (*P* < 0.05) and F/B ratio (*P* < 0.05) compared to APAP group [24]. In addition, administration with EUL also decreased *Verrucomicrobia* (*P* < 0.05) and enriched *Actinobacteria* (*P* < 0.05). It suggests that EUL alter the taxonomic abundance of the microbiome.

As shown in Fig. 4E, the top 50 alteration genera in the Ctrl, APAP and APAP + EUL group were identified. Nineteen and nine obviously altered genera were respectively found in APAP versus Ctrl group and APAP versus APAP + EUL group respectively (*P* < 0.05). Comparison between APAP and Ctrl group, showed that the abundance of *Muribaculaceae, Alistipes, Ruminococcus_1* and *Bacteroides* were significantly increased in APAP, while *unclassified_Bacteria, Ruminiclostridium_9, Oscillibacter, Ruminiclostridium, unclassified_Ruminococcaceae, Lachnospiraceae_UCG-006, Roseburia, Lachnoclostridium, unclassified_Rhizobiaceae, Chloroplas, Desulfovibrio, [Eubacterium]_coprostanoligenes_group, [Eubacterium]_oxidoreducens_group, [Eubacterium]_xylanophilum_group* and *unclassified_Clostridiales* were down-regulated. In contrast to APAP group, EUL mediated an obvious increase in the genera of *Oscillibacter, Ruminiclostridium, Lachnospiraceae_UCG-006, Lachnoclostridium, Bacteroides, Parabacteroides, Lactobacillus, Desulfovibrio* and* [Eubacterium]_xylanophilum_group.*

LEfSe analysis was achieved to filter the specific biomarkers of intestinal microbial taxa related to different interventions. According to the LDA value > 2.5, *P* < 0.05, a total of 73 significantly altered taxa from phylum to genus level were found as key discriminants among the Ctrl group, APAP group and APAP + EUL group (Fig. 4F). The Ctrl group shown a predominance of *Lachnospiraceae_UCG_007, Ruminiclostridium_9, Oscillibacter, Enterobacter, Chloroplast, Lachnospiraceae_UCG_006, Papillibacter, Eubacterium__oxidoreducens_group, Intestinimonas, GCA_900066225, Desulfovibrio, Harryflintia, Lachnoclostridium, Bilophila, Ruminococcaceae_NK4A214_group, Escherichia_Shigella, Family_XIII_UCG_001, Tyzzerella, Angelakisella, Eubacterium__brachy_group, A2, Lachnospiraceae_FCS020_group, Butyricimonas,* and *Dubosiella* at the genus level. In contrast, the APAP group characterized the genera, involving *Muribaculaceae, Erysipelatoclostridium, NS3a_marine_group, Polaribacter_4, Alistipes, Ruminococcus_1,* and *Muribaculum*. After treatment with EUL, *Ruminiclostridium_1*, *Family_XIII_AD3011_group, Lactobacillus, Rikenella, Enterorhabdus, Bacteroides, Parabacteroides, Ruminococcaceae_UCG_010, Prevotellaceae_UCG_001, Prevotellaceae_NK3B31_group,* and *Eubacterium__nodatum_group* were identified as microbial markers*.* Therefore, these bacteria could be considered as biomarkers for EUL-treated mice and become intestinal indicators to relieve APAP-induced liver injury. Particularly, among of them, 6 microbial genera, containing *Desulfovibrio, [Eubacterium]_xylanophilum_group, Oscillibacter, Ruminiclostridium, Lachnospiraceae_UCG-006, Lachnoclostridium*, were reversed by EUL responding to APAP treatment. ROC curve analyses revealed that the AUC of above 6 bacteria is greater than 0.75 (Fig. S5).

### EUL relieves APAP-induced microbial BAs dysmetabolism

**Fig. 5:**
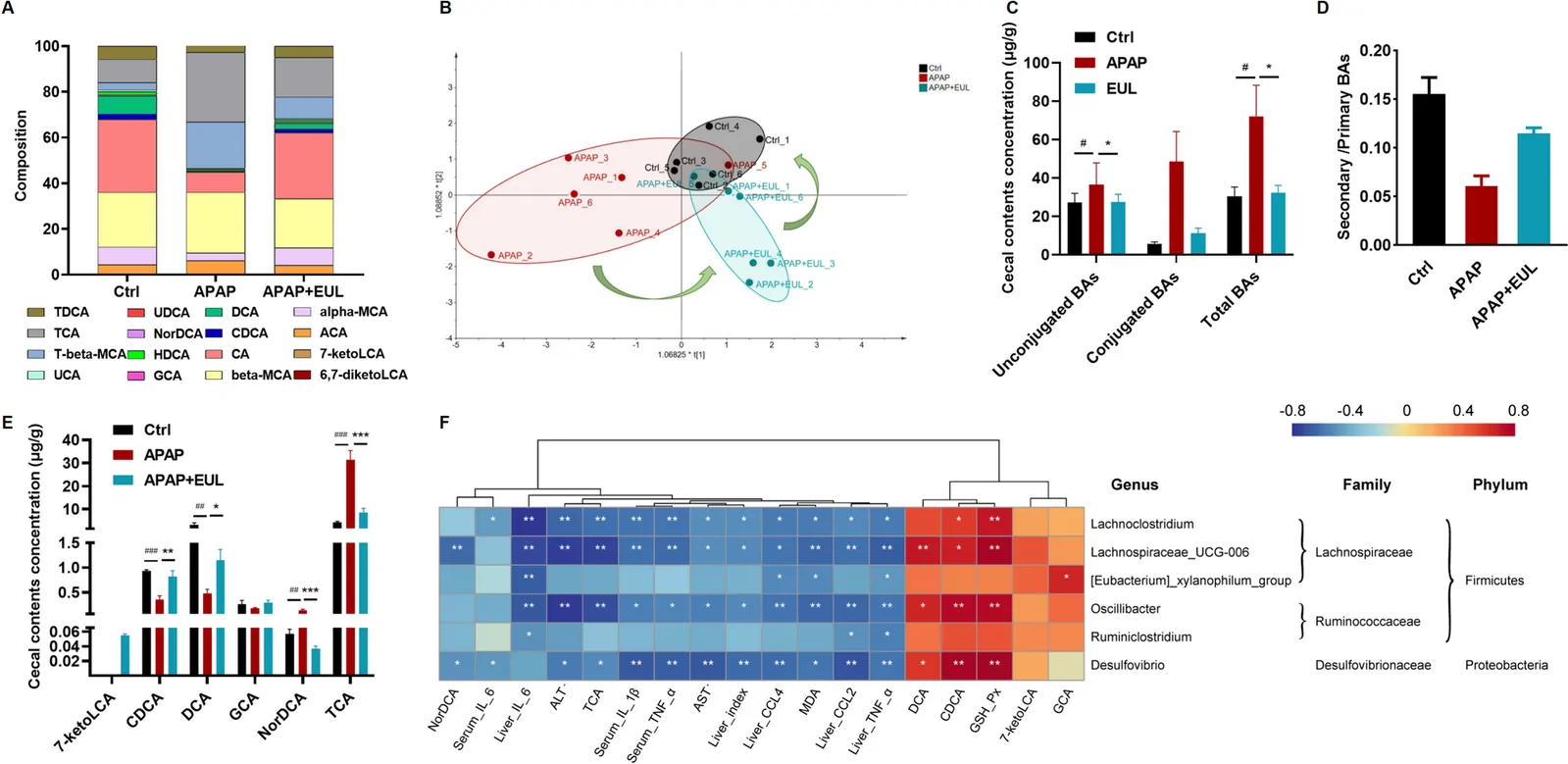
EUL attenuated the disturbance of microbial bile acids (BAs) metabolism in AILI mice. EUL, flavonoids from *Eucommia ulmoides* Oliv. Leaves; AILI: acetaminophen-induced acute liver injury. **A** Composition of bile acid pool in cecal contents. **B** OPLS-DA score plots discriminating the microbial BAs profiles of three groups. **C** The concentration of unconjugated BAs, conjugated BAs and total BAs in cecal contents. **D** Ratios of secondary/primary BAs in cecal contents. **E** The concentration of potential BAs biomarkers in the three groups. **F** Heatmap showing the correlation between microbial BAs, biochemical indicators and gut microbiota. All analyses of were based on a high dose of EUL (100 mg/kg). Data are expressed as the mean ± SEM and analyzed by student t test (n = 6). ^#^*P* < 0.05, ^##^*P* < 0.01, ^###^*P* < 0.001, compared with Ctrl group; ^*^P < 0.05, ^**^P < 0.01, ^***^P < 0.001, compared with APAP group

It has been reported that gut microbiota participated in the regulation of the host organism under the disease situation by influencing host metabolism, such as bile acids metabolism, lipid metabolism, amino acid metabolism [12, 16]. Bile acids (BAs) are considered as endogenous regulators of host co-metabolism [78]. Moreover, the excess of APAP leads to imbalance of BAs cycle, which further affects liver injury and liver regeneration [7]. In this section, BAs metabolomics in cecal contents was observed in each group. A total of 16 BAs across all treated groups (Fig. 5A). Orthogonal Partial Least Squares-Discriminant Analysis (OPLS-DA) exhibited significant clustering of the BAs in the Ctrl, APAP, and EUL-treated groups (Fig. 5B). Noticeably, the BAs metabolite cluster of the EUL group was much closer to that of the Ctrl than the APAP group, suggesting that the efficacy of EUL may partly depend on its role in balancing microbial BAs. Moreover, the levels of total BAs (*P* < 0.05), especially the unconjugated BAs (*P* < 0.05), were higher in APAP group compared with those in the Ctrl and were restored in the EUL-treated group (Fig. 5C). Compared with APAP group, the EUL and Ctrl groups also trended to have lower levels of conjugated BAs and increased the proportion of secondary BAs (Fig. 5D), which in turn decreased inflammation cytokines, leading to the integrity of the gut barrier [70].

Six metabolites (including 7-ketoLCA, DCA, GCA, CDCA, DorDCA and TCA) with VIP > 1.0 were considered as potential biomarkers in the three groups (Tab.S3) [28]. Interestingly, APAP intervention dramatically declined the levels of CDCA (*P* < 0.01), which is natural agonists of FXR [25], while EUL treatment markedly elevated that levels. Furthermore, EUL administration led to an up-regulation in the levels of 7-ketoLCA (*P* < 0.05), DCA (*P* < 0.05) and GCA (*P* > 0.05), and a down-regulation in the levels of DorDCA (*P* < 0.001) and TCA (*P* < 0.001) compared with that in the APAP group (Fig. 5E). ROC curve analyses suggested that the AUC values of 7-ketoLCA and GCA were greater than 0.5, and that value of other biomarkers were equal to 1 (Fig. S4B).

To explore the relationship between gut dysbiosis, changes of biochemical indicators and cecal contents BAs during APAP-induced liver injury, spearman’s correlation analysis was performed (Fig. 5F). In the phylum *Firmicutes*, family *Lachnospiraceae* particularly genera *Lachnoclostridium* and *Lachnospiraceae_UCG-006,* as well as family *Ruminococcaceae*, such as genus *Oscillibacter*, were positively correlated with CDCA, DCA and GSH-Px, and were negatively correlated with TCA, ALT, AST, MDA, liver index, inflammatory factors and chemokines in liver (IL-6, TNF-α, CCL4, CCL2), inflammatory factors in serum (IL-1β, TNF-α). Genus *[Eubacterium]_xylanophilum_group* show positively correlated with GCA and was negatively correlated with MDA and hepatic IL-6, CCL4, TNF-α. Thus, by increasing the abundance of these bacteria belonging to *Firmicutes*, EUL supplementation might exert effective effects on alleviating APAP-induced liver injury, reducing inflammation and oxidative damage and balancing BAs metabolism. In addition, the phylum *Proteobacteria*, genus *Desulfovibrio* was positively correlated with CDCA, DCA and GSH-Px, and were negatively correlated with TCA, NorDCA, ALT, AST, MDA, liver index, inflammatory factors and chemokines in liver (TNF-α, CCL4 and CCL2), inflammatory factors in serum (IL-6, IL-1β, TNF-α). Taken together, EUL administration could attenuate disordered microbial BAs metabolism in AILI mice, that is partly linked to reshape gut microbiota.

### EUL attenuates APAP-induced acute liver injury in a gut microbiota-dependent manner

Antibiotic cocktails were employed to study the relationship between liver protection of EUL and intestinal microecology in APAP induced acute liver injury mice. Clustering tree analysis revealed greatly differences in the gut microbiota composition in APAP + ABX group (compared with APAP group) and APAP + ABX + EUL group (compared with APAP + ABX group) (Fig. 4). After antibiotic cocktails intervention, the APAP + ABX treated mice showed significantly down-regulation of microbial community diversity (*P* < 0.05) compared with the baseline values (Fig. S6A). At the phylum level, the proportion of *Firmicutes*, *Bacteroidetes,* and *Verrucomicrobia* have been dramatically declined, while the abundance of *Proteobacteria*, *Actinobacteria Patescibacteria* and *Cyanobacteria* were obviously elevated in antibiotic-treated mice (including APAP + ABX + EUL group and APAP + ABX group) (Fig.S6 A). At genus level, most bacteria regulated by EUL were blocked by antibiotics cocktails (Fig. 4E).

Consistent with the gut microbiota change, the protective effect of EUL against APAP hepatotoxicity was disturbed when the antibiotic cocktails inhibited the intestinal microbiota. As the data shown, ABX + EUL group remarkable increased the activity of GSH-Px (*P* < 0.01) in hepatic tissue and significantly declined the liver levels of TNF-α (*P* < 0.05), IL-6 (*P* < 0.05), CCL3 (*P* < 0.05), compared with those in APAP + ABX group (Fig. S6B-Q). However, no significant difference was monitored in the liver index; serum levels of ALT, TNF-α, IL-1β, IL-6, CXCL10, CCL4, CCL2; liver levels of MDA, CXCL10, CCL4, CCL2, between the APAP + ABX group and APAP + ABX + EUL group (Fig.S6 B-Q). The above results indicate that EUL-induced hepatic protection in APAP-caused acute liver injury could be depended on the modulation on gut microbiota.

Similarly, BAs metabolomics profiling of cecal contents indicated that the regulation in the concentrations of BAs by EUL treatment was blocked by antibiotic intervention (Fig. S7).

These findings demonstrated that the antibiotics broken the protective effect of EUL in protecting the liver, relieving intestinal flora disorder and regulating fecal bile acid metabolism. Therefore, we suggested that EUL regulate bile acids metabolism against APAP hepatotoxicity in a gut microbiota-dependent manner.

### The hepatic protection of EUL is transferable by fecal microbiota transplantation

**Fig. 6:**
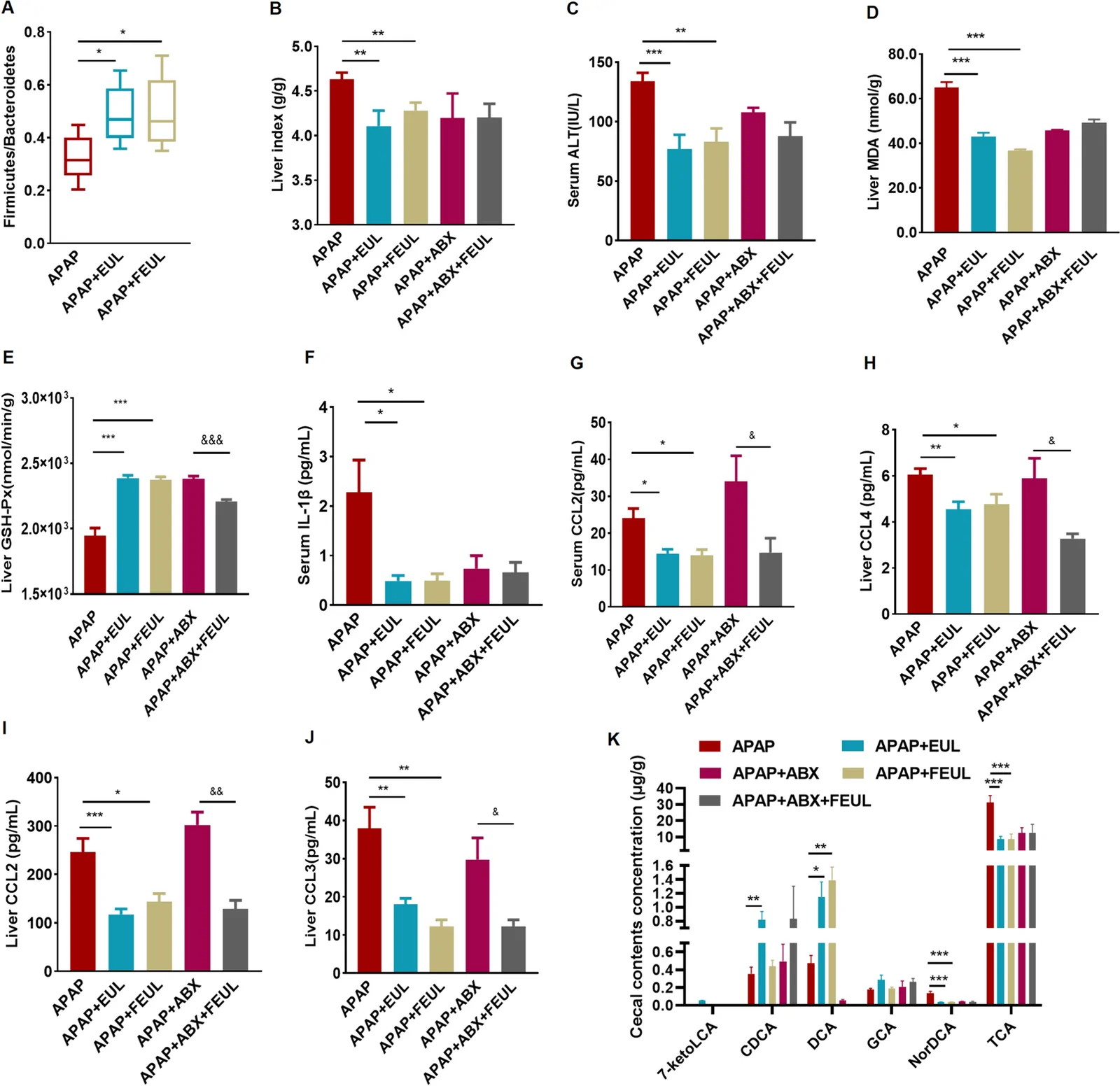
The hepatic protection of EUL is transferable by fecal microbiota transplantation (FTM). EUL, flavonoids from *Eucommia ulmoides* Oliv. Leaves. **A** Firmicutes-to-Bacteroidetes (F/B) ratio; Data are expressed as the median (min–max) and analyzed by student t test (n = 7). **B** Liver index. **C** Serum ALT. **D** Liver MDA. **E** Liver GSH-Px. **F** Serum IL-1β. **G** Serum CCL2. **H** Liver CCL4. **I** Liver CCL2. **J** Liver CCL3. Data in **B**–**J** are expressed as the mean ± SEM and analyzed by ANOVA (n = 7). **K** The concentration of potential BAs biomarkers in different groups; Data are expressed as the mean ± SEM and analyzed by student t test (n = 6). All analyses of were based on a high dose of EUL (100 mg/kg). ^#^*P* < 0.05, ^##^*P* < 0.01, ^###^*P* < 0.001, compared with Ctrl group; ^*^P < 0.05, ^**^P < 0.01, ^***^P < 0.001, compared with APAP group. The APAP and EUL data in this panel are identical to those shown in Fig. 3 because the same experimental cohort was used

To further illustrate the beneficial effects of EUL mediated by the gut microbiota and its influence on the host bile acids, we transferred the microbiota from APAP + EUL (donor mice) group to APAP + FEUL (recipient mice) group mice. At the phylum level, the APAP + FEUL group retained the abundance advantage of *Firmicutes* (Fig. S6A and Fig. 6A). At the genus level, similar to APAP + EUL group, APAP + FEUL group significantly reversed the levels of differential bacteria, including *Oscillibacter* and *Desulfovibrio*, that were decreased by APAP (Fig. 4E).

EUL receivers (APAP + FEUL) showed substantially decreased liver index (*P* < 0.01), and reduced serum levels of ALT (*P* < 0.01), IL-1β (*P* < 0.05), CCL2 (*P* < 0.05), as well as lower levels of liver MDA (*P* < 0.001), CCL4 (*P* < 0.05), CCL3 (*P* < 0.01), CCL2 (*P* < 0.05), as compared to APAP group (Fig. 6C–J). Moreover, APAP + FEUL also developed improved GSH-Px (*P* < 0.001) in liver as compared to APAP group (Fig. 6E). APAP + FEUL treated mice also maintained a downward trend in serum levels of CCL4 and liver levels of TNF-α, IL-6, but there was no significant difference (Fig. S7C–E).

Treatment with FEUL also reserved the increasing the concentration of DCA (*P* < 0.001), and maintained the decreasing of DorDCA (*P* < 0.001) and TCA (*P* < 0.001) (Fig. 6K).

In particular, consistent with the APAP + ABX + EUL group, the antibiotics broken the protective effect of APAP + ABX + FEUL in protecting the liver, relieving intestinal flora disorder (Fig. 6C–J) and regulating fecal bile acid metabolism (Fig. S7A).

Together, these results demonstrate that the bacterial transplanted mice (APAP + FEUL) reproduce the functions of liver protection, regulation of microorganisms and metabolism observed in donor mice (APAP + EUL), which partly declares the hepatic protection of EUL mediated by gut microbiota.

### EUL alleviate APAP-induced liver injury via pathways involving gut microbiota-BAs-FXR-FGF15

**Fig. 7:**
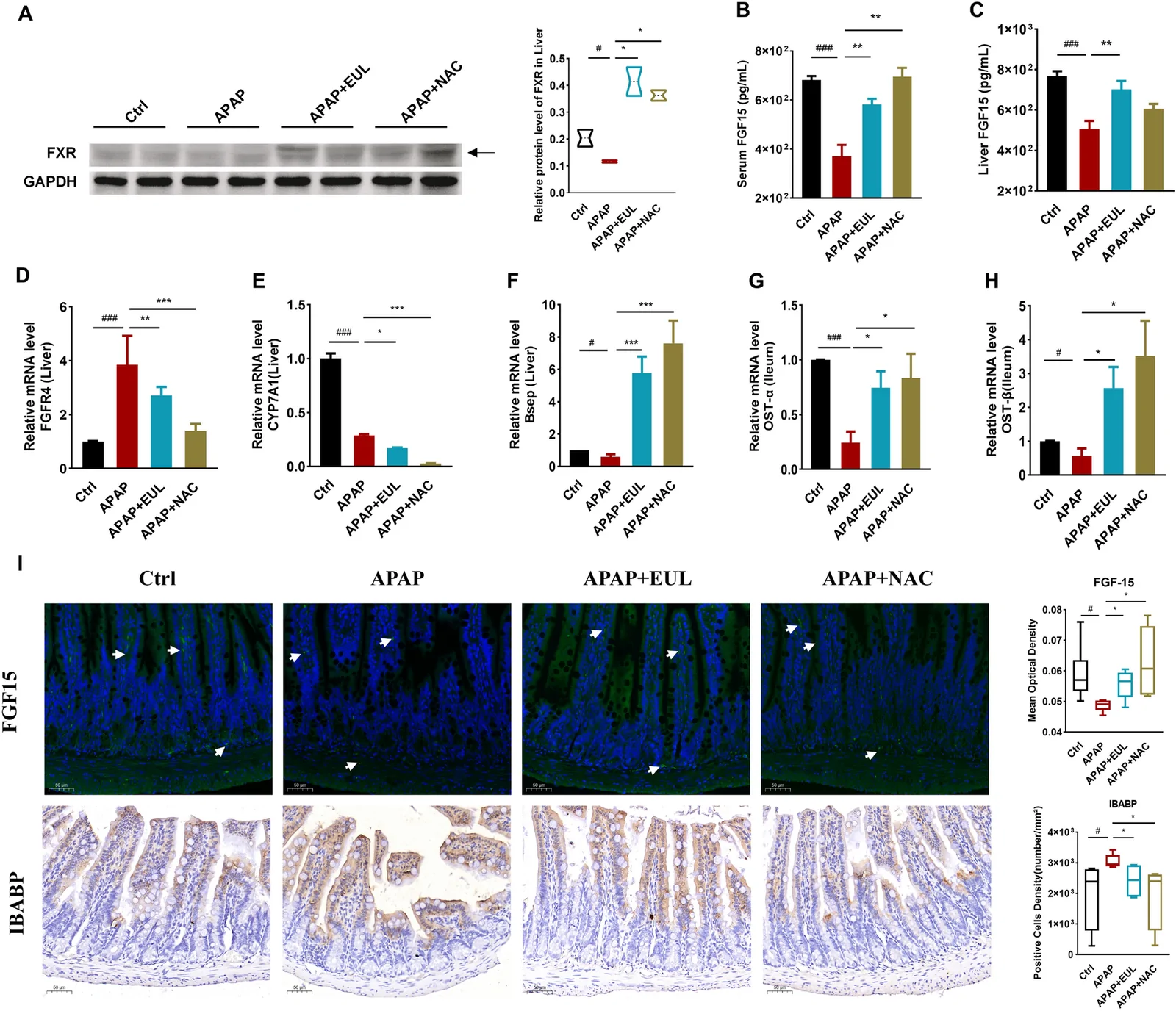
EUL alleviate AILI through pathways involving gut microbiota-BAs-FXR-FGF15. EUL, flavonoids from *Eucommia ulmoides* Oliv. Leaves; AILI: acetaminophen-induced acute liver injury. **A** The relative expression levels of FXR proteins in ileum tissues (n = 3). **B**, **C** FGF15 levels in serum and liver tissues (n = 6). **D**–**F** FGFR4, CYP7A1 and Bsep mRNA expression levels in liver tissues. **G**, **H** OST-α and OST-β mRNA expression levels in ileum tissues (n = 7). **I** Representative images of FGF15 and IBABP immunostaining in ileum tissues, scale bar = 50 µm (n = 3). Data in **A** and **I** are expressed as the median (min–max) and analyzed by student t test. Data in (B-H) are expressed as the mean ± SEM and analyzed by ANOVA. All analyses of were based on a high dose of EUL (100 mg/kg). ^#^*P* < 0.05, ^##^*P* < 0.01, ^###^*P* < 0.001, compared with Ctrl group; ^*^P < 0.05, ^**^P < 0.01, ^***^P < 0.001, compared with APAP group

Hepatic secretion and intestinal absorption are two critical parts of enterohepatic circulation of BAs. In order to further survey the approach of intestinal bacteria regulating BCs metabolism, targets closely involved to BCs synthesis, reabsorption and efflux were detected. Previous reports have shown that the related action of FXR and FGF19/15 (human FGF-19, mice FGF-15) is the crucial link for gut-liver axis in regulating BAs synthesis and BAs pool size [20]. We further examined the protein expression of FXR and FGF15 in ileum by WB and immunofluorescence, respectively. Compared with APAP group, the relative protein levels of FXR in ileum were up-regulated by APAP + EUL (*P* < *0.05*) (Fig. 7A). FGF15 immunostaining in APAP + EUL group was similar to that of the Ctrl group, while the APAP group showed lower level of FGF15 immunostaining (*P* < 0.05) (Fig. 7I). Similarly, the circulating concentration of FGF15 in both serum and liver (*P* < 0.01), detected by ELISA, was increased in the APAP + EUL treatment groups compared with APAP group (Fig. 7B and C). As the information receiver of FGF15 in liver, the expression of FGFR4 mRNA were elevated in APAP + EUL group as compared to APAP group (*P* < 0.01) (Fig. 7D). We also observed markedly declined expression of FGFR4 target gene, such as BAs synthesis gene CYP7A1 (*P* < 0.05) after APAP + EUL treatment (Fig. 7E). Moreover, the mRNA expression of hepatobiliary bile salt export pump (Bsep) (*P* < 0.001) was elevated after treated with APAP + EUL (Fig. 7F). Bsep mediates the efflux of BAs from hepatocytes, which plays an important role in maintaining the homeostasis of BAs [53]. APAP + EUL group also showed a tendency to reduce the positive rate of CK19 staining, resulting in down-regulated bile duct proliferation, which benefits for the AILI recovery (Fig. S8A) [4]. The organic solute transporter-α/β (Ost-α/β) and ileal bile acid binding protein (IBABP) regulated by FXR are responsible for the enterohepatic circulation of BAs and Ost-α/β plays a defensive role in ileum epithelium injury induced by BAs accumulation [5, 17]. In our study, the levels of IBABP immunostaining (Fig. 7I) (*P* < 0.05) and Ost-α/β mRNA expression (*P* < 0.05) in ileum were markedly increased in APAP + EUL treatment compared with the APAP group (Fig. 7G and H).

**Fig. 8:**
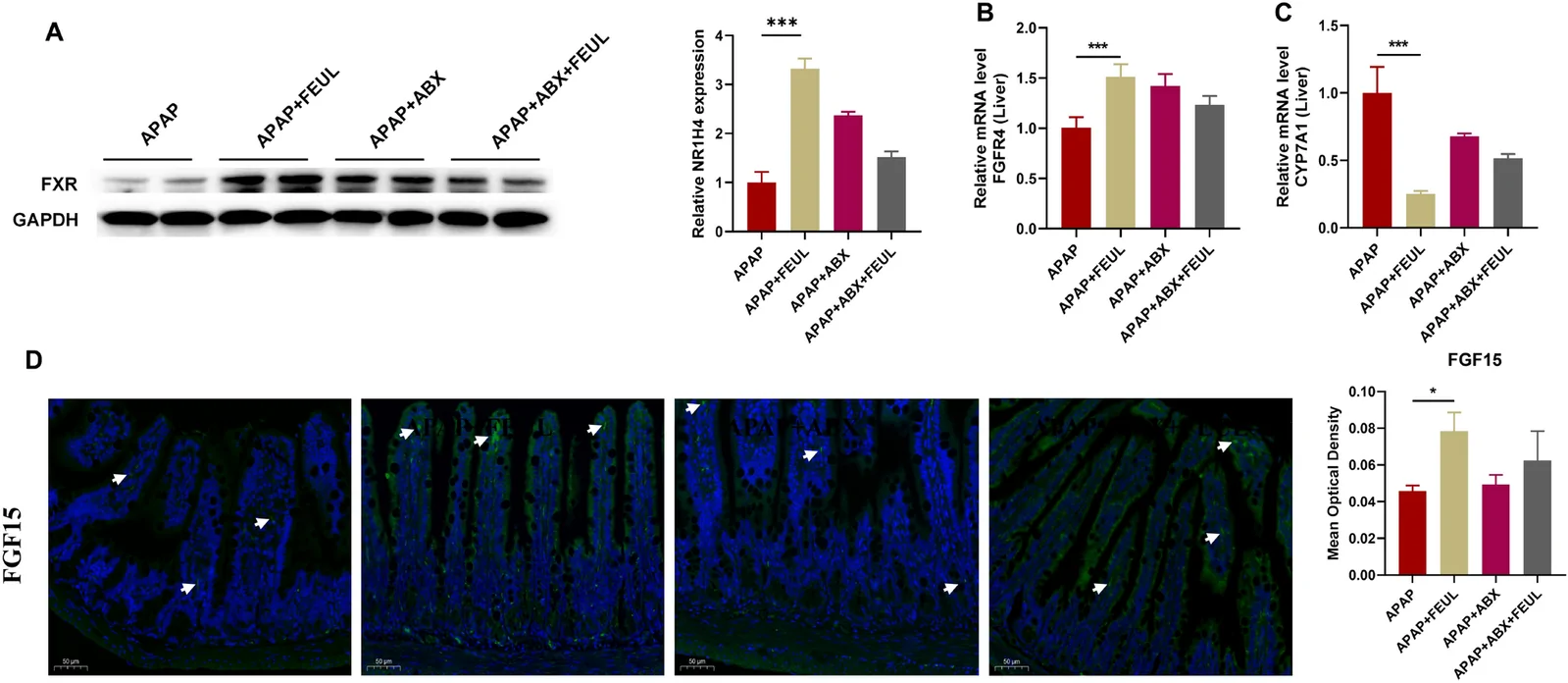
FEUL alleviate AILI. **A** The relative expression levels of FXR proteins in ileum tissues (n = 3). **B**, **C** FGFR4 and CYP7A1 mRNA expression levels in liver tissues (n = 7). **D** Representative images of FGF15 immunostaining in ileum tissues, scale bar = 50 µm (n = 3). Data are expressed as the mean ± SEM and analyzed by ANOVA. ^*^P < 0.05, ^**^P < 0.01, ^***^P < 0.001, compared with APAP group

Moreover, ELISA analysis showed that FEUL treatment increased FGF15 levels in both serum and liver compared with the APAP group (Fig. S8B–E). Consistently, immunofluorescence staining demonstrated increased FGF15 expression in the ileum following FEUL treatment (Fig. 8A). In ileum, FEUL markedly upregulated FXR protein expression (Fig. 8B, *P* < 0.05) and FGFR4 mRNA expression (Fig. 8C, *P* < 0.001), while suppressing CYP7A1 mRNA levels (Fig. 8D, *P* < 0.001). However, compared with the APAP + ABX group, the levels of FGF15 in both serum and liver were not markedly restored in the APAP + ABX + EUL or APAP + ABX + FEUL groups (Fig.S8B-E). Similarly, the regulatory effects of FEUL on hepatic FXR, FGFR4, and CYP7A1 were largely attenuated after antibiotic cocktail treatment.

The results of mRNA and protein analysis indicated that EUL may ameliorate the disturbance of fecal bile acid metabolism by affecting the FXR-FGF15 signaling, which is partly influenced by gut microbiota.

## Discussion

APAP overdose-induced liver injury (AILI) is the most frequent reason for acute liver failure in Asian, American and European countries [71]. The formation and development of AILI are complex, which are relevant to hepatic inflammation and oxidative stress, hepatocyte apoptosis, gut microbial imbalance, cholestasis, etc. [1, 8, 21, 67, 94]. Accumulating research reports that the flavonoids from *Eucommia ulmoides* Oliv. Leaves (EUL) and their extracts show the prominent anti-inflammatory and antioxidant activity, which may provide a new and promising liver protection strategy [14, 23, 35, 97]. Tn clinical and animal studies, the pathogenesis of AILI is mainly characterized by hepatic dysfunction, such as elevated transaminase levels, and increased oxidative stress and inflammation in the liver [58, 76, 81]. In our study, it was found that EUL supplementation obviously relieved AILI by reducing serum AST and ALT levels, attenuating hepatic inflammation and oxidative stress, restoring liver histological changes, as well as improving intestinal barrier integrity. In addition, its positive effect was associated with reshaping the composition of gut microbiota and regulating bile acid metabolism and accompanied by activation of the FXR pathway at gut-liver axis.

In this study, the extraction and purification process of flavonoids in EUL was optimized. Quercetin 3-O-sambinoside, rutin and isoquercetin are the active components of EUL with antioxidant properties, and their enrichment may contribute to the down-regulation of liver MDA and GSH-Px in this study [11, 98]. Rutin and isoquercetin also reported to prevent AILI by suppressed the levels of ALT, AST, TNF-α, IL-1β, and IL-6, that are confirmed our results [40, 69, 85]. Astragalin significantly increased the expression of intestinal ZO-1 and Occludin, suggesting an advantage in improving the intestinal barrier and regulating the intestinal microbiota, which is consistent with our observations [60]. Altogether, the purification of the above components provides hepatoprotective potential for EUL to alleviate AILI.

Disturbed gut microbiota has been reported to contribute to susceptibility in animal models of APAP-induced hepatotoxicity, with the most commonly reflected in declined microbiota diversity, lack of beneficial bacteria, and barrier impairment [24, 67]. It may be attributed to that the dysregulated gut microbiota accelerates intestinal inflammation, aggravates intestinal permeability, and allows bacterial escape from the damaged intestinal mucosa leading cytokine-mediated liver injury [66, 74]. As expected, EUL administration up-regulated the abundance of beneficial bacteria in the APAP-induced liver injury mice, while the alleviating effects of EUL on the symptoms of hepatic damage were completely disrupted in antibiotic cocktail-treated mice, indicating that the gut microbiota take a vital role on hepatic protection of EUL. Moreover, fecal microbiome transplantation (FMT) from EUL-supplemented mice also showed reduced hepatic damage in recipient mice, which partly supported that the therapeutic function of EUL depends on reshaping the gut microbiota. Consistent with previous studies, at the phylum level, APAP mice developed significantly decreased abundance of *Firmicutes*, whereas the *Bacteroidetes* was increased, resulting to a lower F/B ratio [13, 24]. By contrast, we found supplement with EUL reversed above changes, that are confirmed with previous studies [96]. Noticeably, high levels of *Firmicutes* in the gut have been reported to be associated with reduced systemic inflammation [64]. At family levels, EUL-treated mice obviously elevated the abundance of *Ruminococcaceae* and *Lachnospiraceae*, which are known to strengthen intestinal barrier and improve intestinal inflammation by participating in metabolic regulation [9, 42, 80]. Literature supports that *Lachnospiraceae* deficiency amplifies the risk of AILI [67]. In addition, as the 7α-dehydroxylating bacteria, *Lachonospiraceae* and *Ruminococcaceae*, are responsible for converting primary bile acids (CA and CDCA) into secondary bile acids (DCA and LCA) [19]. Therefore, the increased proportion of secondary bile acids (including DCA, NorDCA and 7-ketoLCA) in the cecal contents of EUL-treated mice can be partly due to reshape the intestinal dysbiosis to up-regulate the abundance of *Lachonospiraceae* and *Ruminococcaceae*. Based on the improved intestinal bacteria which associated with AILI, the correlation analysis with spearman strategy verified that there is a remarkable correlation between gut microbiota and hepatic inflammatory and oxidative stress. For instance, at the genus levels, EUL enriched the abundance of *Lachnoclostridium*, *Lachnospiraceae_UCG-006*, *Oscillibacter* and *Desulfovibrio*, which were positively correlated with GSH-Px concentration and negatively correlated with MDA, IL-6, TNF-α, CCL4, CCL2 levels in the serum or liver. Consistent with our findings, Gou et al. found that elevated levels of *Lachnospiracae_UCG-006* were negatively correlated with serum IL-6, liver AST and ALT levels, which played a protective role in acute liver injury and inflammation [26]. *Desulfovibrio* has recently been proven not to be always associated with adverse health effects, the research suggested that tea polyphenol and epigallocatechin gallate promoted the abundance of *Desulfovibrio* alleviated liver fat accumulation and protected liver function via remodeling gut metabolism [82]. In addition, pretreatment with EUL also promoted the abundance of *Ruminiclostridium* and *[Eubacterium]_xylanophilum_group*. Both of them are reported to have mucosal protective, nutritional and intestinal epithelial protective functions, which are beneficial to protect liver injury [32, 55, 104]. Enriched *Lachnoclostridium* and *Oscillibacter* possess similar functions [90, 100]. These altered bacteria indicate that EUL has the potential functions in inhibiting intestinal inflammation, maintaining the integrity of the intestinal barrier, reshaping gut microbiota and regulating microbial BA metabolism, all of which contribute to the alleviation of AILI.

The maintenance of BAs homeostasis in the gut-liver axis plays an indispensable role in repairing liver Injury and regeneration following APAP overdose [7, 21]. Both animal and clinical studies have demonstrated that the elevation of bile acids, especially conjugated BAs, is associated with APAP exposure and toxicity, and promoting the excretion of BAs is beneficial to ameliorate hepatotoxicity [1, 21, 38, 83]. Our results revealed that the total BAs, particularly conjugated BAs (such as TAC), in cecal contents sample was obviously declined with EUL stimulation, which is in line with previous data. Abnormally elevated in TCA was not only proved to be responsible for the ileum permeability, leading to intestinal permeability leakage, but also considered to trigger hepatic stellate cell activation and promote systemic inflammation and liver fibrosis [50, 73, 88]. Furthermore, after EUL administration, the hepatic BSEP and intestinal OST-α/β in the mRNA levels were up-regulated, which demonstrated that the efflux of BAs in both liver and ileum was promoted [6]. OSTα/β has a stronger affinity for unconjugated BAs, while BSEP preferentially transports conjugated BAs [72, 88]. Thus, we speculated that it might be the result of EUL promoting the excretion of BAs. Meanwhile, we found that supplementation of EUL changed the proportion of primary bile acids (including CDCA, GCA, TCA) and secondary bile acids (including 7-ketoLCA, NorDCA and DCA). The absence of secondary bile acids has been shown to contribute to the exacerbation of intestinal and liver inflammation [22, 70]. Gillard et al. found that supplementation with secondary BAs, such as DAC, contribute to regulate the composition of BA in portal vein blood, increase FXR signal, reduce macrophage infiltration, repair inflammation and oxidative stress in hepatic [22]. DCA and LCA is the most abundant gut secondary bile acids, which has been demonstrated to suppress multiple chemokines (such as CCL2, CCL5, and CXCL10) and cytokines related to inflammation (such as IL-1β, IL6 and TNF-α) [70, 92]. In addition, DCA is a recognized TGR5/GPBAR1 agonist. TGR5 activation has been reported to regulate macrophage inflammatory signaling through cAMP/PKA-related pathways and to suppress inflammatory mediators, including pathways linked to NF-κB and NLRP3 activity [18]. Therefore, the observed changes in DCA may have implications beyond the FXR/FGF15 axis, especially in relation to hepatic inflammation and macrophage-associated immune regulation. Recent studies show that TCA and GA are potential biomarkers for DILI [62]. In addition, we found that EUL significantly increased the levels of CDCA, which was is the main endogenous ligands of FXR. FXR is mainly expressed in the intestine and liver, and its activation contributes to the reduction of AILI, that is partly attributed to its effects on reducing inflammation and oxidative stress [2, 91, 102]. Importantly, the activation of intestinal FXR is involved in maintaining the BAs homeostasis at gut-liver axis [44]. Ileal FXR inhibits hepatic FGFR4 expression by inducing an increase in gut-derived FGF15, thereby decreasing the level of hepatic BAs synthase CYP7A1 [33, 63]. Therefore, supplementing EUL may have the potential to reduce the synthesis of BAs. Kim et al., also found that inhibition of CYP7A1 in the liver requires ileal FXR activation, not the hepatic one [44]. Additionally, FXR also mediate the expression of OST-α/β and IBABP, which contribute to bile acid homeostasis [41, 87, 89]. In our study, EUL not only restored the levels of changed BAs but also activated FXR-FGF15 signaling in the gut-liver axis of AILI mice. Based on these results, we speculated that supplementation of EUL may be a potential FXR activator, or it indirectly activate the FXR-FGF15 pathway to alleviate AILI through regulating the metabolism of BAs, which still needs further research.

Interestingly, we found that the APAP + FEUL group preserved the regulation of EUL on microbial BAs metabolism and BAs enterohepatic circulation related pathways, while antibiotic intervention limited the metabolic regulation of APAP + ABX + EUL and APAP + ABX + FEUL groups. Moreover, Pearson correlational analysis in this study confirmed that the abundance of genus bacteria (*Lachnospiraceae_UCG-006*, *Oscillibacter* and *Desulfovibrio*) were positively correlated with CDCA and DCA, but negatively correlated with TCA and NorDCA. It might suggest that reshaping intestinal homeostasis was an indispensable approach for EUL to alleviate AILI, and its regulation of microbial BAs was partly influenced by gut microbiota.

Our research also has some limitation. First, APAP-induced acute liver injury is a multifactorial process involving intricate interactions among diverse mechanisms and signaling pathways, whereas only selected aspects were examined here. Accordingly, the full pathological complexity of AILI and the complete protective mechanism of EUL remain to be further clarified. Second, bile acid analysis was confined to colonic contents, which mainly reflect local microbiota-dependent transformations, especially secondary bile acid metabolism. This approach does not fully represent systemic bile acid homeostasis, which is jointly regulated by hepatic synthesis, biliary secretion, intestinal reabsorption, and enterohepatic circulation. Future studies incorporating systemic and hepatic bile acid profiling will be required to define the overall impact of EUL on bile acid metabolism more comprehensively. Third, intestinal barrier integrity was assessed mainly by indirect histological and immunohistochemical evidence, without direct functional assays such as intestinal permeability testing or serum LPS measurement. Fourth, although our data support the involvement of the gut microbiota-BAs-FXR-FGF15 axis, direct mechanistic validation in FMT recipient mice was not fully performed. Specifically, pathway-related target changes were not further examined in the FMT groups. Finally, although pretreatment strategies may be difficult to translate directly into clinical practice because drug-induced liver injury is often unpredictable in humans, the mechanistic insights provided by this study may still offer a valuable basis for future preventive and therapeutic investigations.

## Conclusion

**Fig. 9:**
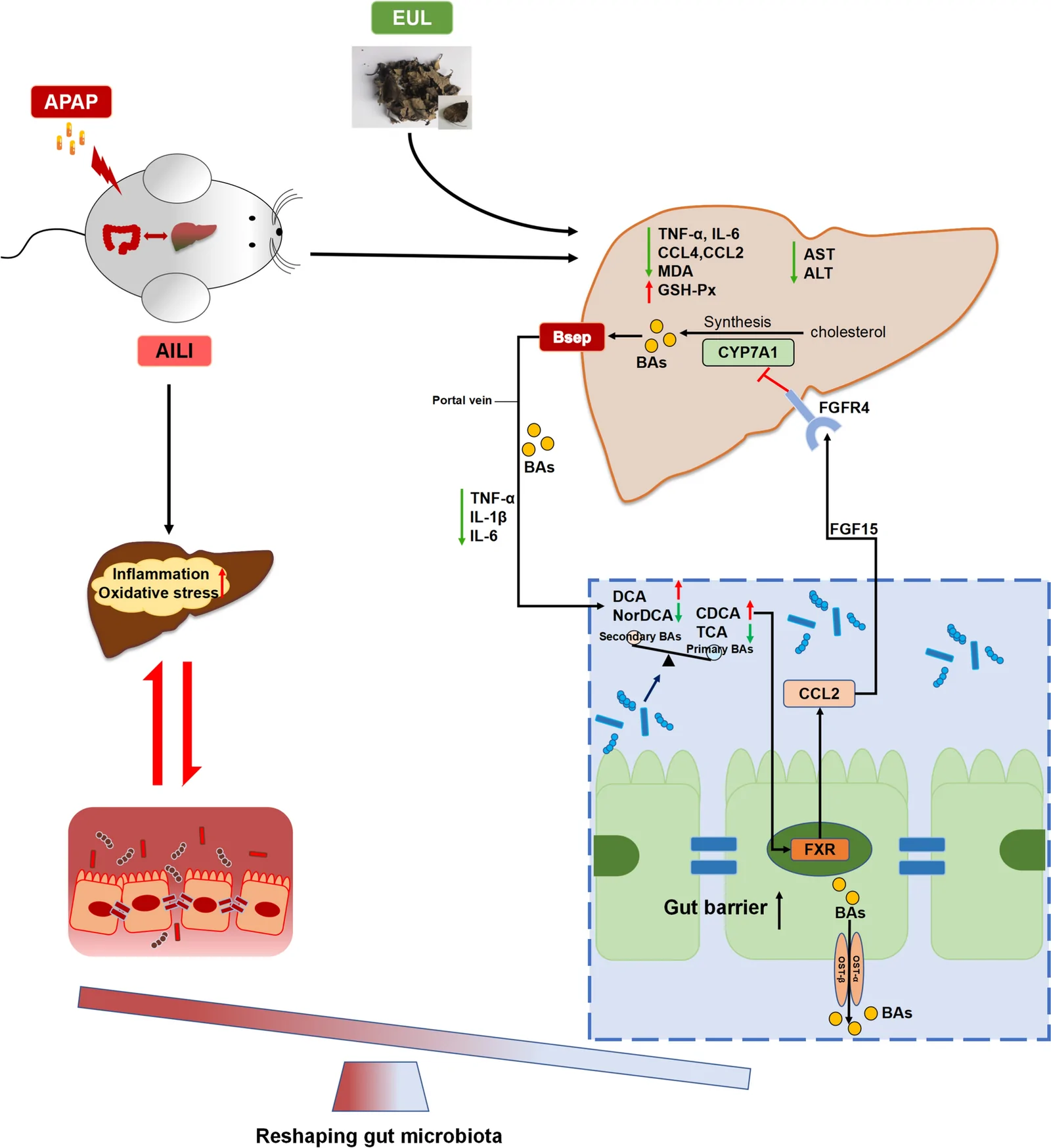
EUL alleviate AILI by reducing hepatic inflammation and reshaping gut microbiota composition, which further activate the FXR-FGF15 pathway to restore the altered microbial BAs metabolism. EUL: flavonoids from *Eucommia ulmoides* Oliv. Leaves; AILI: acetaminophen-induced acute liver injury

Taken together, we investigated the mechanism of EUL in relieving AILI mice combined with 16S rRNA technology and bile acid-targeted metabolomics. This study revealed that EUL supplementation could reduce liver inflammation and oxidative stress to alleviate the hepatotoxicity induced by APAP. In addition, EUL administration possesses the positive effects in reducing intestinal inflammation, maintaining the integrity of the intestinal barrier, reshaping gut microbiota composition and restoring altered microbial BAs metabolism. The mechanism of EUL improving AILI may be closely associated to the intestinal microbiome-BAs-FXR signaling in gut-liver axis (Fig. 9). Our findings provide an alternative strategy for treating AILI, and also show novel insights for EUL in development and utilization.

## Supplementary Information

Supplementary material 1.

## Data Availability

No datasets were generated or analysed during the current study.

## Abbreviations

APAP: Acetaminophen
AILI: Acetaminophen-induced liver injury
ALF: Acute liver failure
AST: Aspartate aminotransferase
ALT: Alanine aminotransferase
BAs: Bile acids
CYP: Cytochrome P450 enzyme
CDCA: Chenodeoxycholic acid
DCA: Deoxycholic acid
EUL: Flavonoids from *Eucommia ulmoides* Oliv. Leaves
FMT: Fecal microbiome transplantation
FXR: Farnesoid X Receptor
FGF15: Fibroblast growth factor-15
FGFR4: Fibroblast growth factor receptor-4
IL-1β: Interleukin-1β
IL-6: Interleukin-6
GSH-Px: Glutathione peroxidase
NorDCA: Norcholic acid
NAPQI: N-acetyl-p-benzoquinone imine
NAC: N-Acetylcysteine
MDA: Malondialdehyde
TJ: Tight junction
7-ketoLCA: 7-Ketodeoxycholic acid
TNF-α: Tumor necrosis factor-α
16S rRNA: 16S-ribosomal RNA
