# Supplementary material for: Extract from Eucommia ulmoides Oliv. Leaves alleviates APAP-induced acute liver injury via reshaping gut microbiota and attenuating microbial bile acids metabolism disorder
Source: Chin Med. 2026 Oct 14;21:228. doi: 10.1186/s13020-026-01496-9 (PMC13670502; doi:10.1186/s13020-026-01496-9)
Supplement: Supplementary file 1 [file 13020_2026_1496_MOESM1_ESM.docx]

**Supplementary Information**

**Quantitative and qualitative analysis of the major components in water extract of** ***Eucommia ulmoides* Oliv. leaves**

High performance liquid chromatography of quadrupole time of flight mass spectrometry (HPLC-Q-TOF-MS/MS) was employed to characterize a total of 40 compounds in *Eucommia ulmoides* Oliv. leaves (Tab.S1 and Fig.S1). Chromatographic separation was conducted on an Inertsil ODS-2 (4.6×250 mm, 5 μm). Sample injection and the flow rate were set at 10μL and 1 mL/min, respectively. The column temperature and wavelength were 30 °C and 254 nm, respectively. The mobile phase consists of 1% formic water (A) and acetonitrile (B). The gradient elution after optimization is as follows: 0-5min, 5-12% (B); 5-12min, 12-22% (B); 12-38min, 22-10% (B); 38-45min, 40-52% (B). The mass spectrometry analysis was performed on negative ions detection mode with the mass range of 113-1000 m/z, and drying gas was 8L/min, the gas temperature was 300 °C. The compounds were identified by MS/MS information, retention time, literature, mzCloud and ChemSpider databases.

Quantitative analysis of four major flavonoids (quercetin 3-O-sambinoside, rutin, isoquercetin and astragalin) in EUL was performed by high performance liquid chromatography coupled with diode array detector (HPLC-DAD). The gradient elution after optimization is as follows: 0-5min, 10-17.5% (B); 5-10min, 17.5-17.5% (B); 10-17min, 17.5-19% (B); 17-22min, 19-19.2% (B); 22-42min, 19.2-19.2% (B); 42-45min, 19.2-19.3% (B).

**Viability assay**

Cell viability was monitored by CCK8 assay (E-CK-A362, Elabscience, China). Briefly, AML12 cells (CL-0602, Procell, China) were seeded at the density of 2.0×10^5^ cells/well in a 96-well plate. Cells were treated with APAP, EUL or APAP+EUL. After 24 h, CCK8 (10 μL/well) was added and incubated for 3 h. The cytotoxicity was determined by microplate reader (ZY2018030023, PerkinElmer, USA).

**Western Blot**

Total protein was extracted from ileum tissues by radioimmunoprecipitation assay (RIPA) lysis buffer and quantitated using a BCA protein assay kit. Western blotting was performed using the following antibodies: FXR(1:1000, 25055-1-AP; Proteintech, Wuhan, China). The band intensities were quantified using ImagePro Plus 6.0.

**Fig.S1** Base Peak Chromatogram of EUL

**Fig. S2** Composition and hepatoprotective activity of EUL. (A)The extraction and purification process of EUL. (B) EUL inhibits APAP induced toxicity in AML12 cells. (C) 1~500μg/mL EUL shows no toxicity to AML12 cells. (D-F) Exploration of modeling dosage. (G-I) Exploration of the intervention effect of EUL Data are expressed as the mean ± SEM by ANOVA. ^#^*P* < 0.05, ^###^*P* < 0.001 compared with Ctrl group; ^*^*P* < 0.05, ^**^*P* < 0.01, ^***^*P* < 0.001 compared with 10mMAPAP group.

**Fig. S3** Acetaminophen (APAP) treated mice developed higher levels of 3-nitrotyrosine. scale bar=50 µm (n=5). All analyses of were based on a high dose of EUL (100 mg/kg). Data are expressed as the median (min-max) and analyzed by student t test. ^#^*P* < 0.05, compared with Ctrl group; ^*^*P* < 0.05, ^**^*P* < 0.01, compared with APAP group.

**Fig.S4** EUL alleviate ileum injury in AILI mice. EUL, flavonoids from *Eucommia ulmoides* Oliv. Leaves; AILI, acetaminophen-induced acute liver injury. Representative images of ZO-1, occludin and claudin-1 immunostaining in ileum tissue; scale bar=50 µm (n=7); All analyses of were based on a high dose of EUL (100 mg/kg). Data are expressed as the median (min-max) and analyzed by student t test. ^#^*P* < 0.05, ^##^*P* < 0.01 compared with Ctrl group; ^*^*P* < 0.05, ^**^*P* < 0.01 compared with APAP group.

**Fig. S5** ROC curve analysis of (A) intestinal bacteria and (B) bile acids.

**Fig. S6** EUL attenuated AIL in a gut microbiota-dependent manner. AILI, acetaminophen-induced acute liver injury. (A) Bacterial taxonomic profiling at the phylum level. (B) Liver index. (C)Serum ALT. (D) Liver GSH-Px. (E) Liver MDA. (F) Serum TNF-α. (G) Serum IL-1β. (H) Serum IL-6. (I) Serum CXCL10. (J) Serum CCL4. (K) Serum CCL2. (L) Liver TNF-α. (M) Liver IL-6. (N) Liver CXCL10. (O) Liver CCL4. (P) Liver CCL3. (Q) Liver CCL2. All analyses of were based on a high dose of EUL (100 mg/kg). Data are expressed as the mean ± SEM and analyzed by ANOVA (n=7). ^#^*P* < 0.05, ^##^*P* < 0.01, ^###^*P* < 0.001, compared with Ctrl group; ^*^*P* < 0.05, ^**^*P* < 0.01, ^***^*P* < 0.001, compared with APAP group; ^&^*P* < 0.05, compared with APAP+ABX group. The APAP and EUL data in this panel are identical to those shown in Figure 3 because the same experimental cohort was used.

**Fig. S7** EUL relieved microbial BAs dysmetabolism in AILI mice via a gut microbiota-dependent manner. EUL, flavonoids from *Eucommia ulmoides* Oliv. leaves; AILI, acetaminophen-induced acute liver injury. (A) The 3D and 2D modes of OPLS-DA score plots discriminating the microbial BAs profiles in different groups. (B) The concentration of potential BAs biomarkers in different groups; Data are expressed as the mean ± SEM and analyzed by student t test (n=6). (C-E) Liver levels of TNF-αand IL-6, and serum levels of CCL4; Data are expressed as the mean ± SEM and analyzed by ANOVA (n=7). All analyses of were based on a high dose of EUL (100 mg/kg). ^#^*P* < 0.05, ^##^*P* < 0.01, ^###^*P* < 0.001, compared with Ctrl group; ^*^*P* < 0.05, ^**^*P* < 0.01, ^***^*P* < 0.001, compared with APAP group; ^&^*P* < 0.05, ^&&^*P* < 0.01, compared with APAP+ABX group. The APAP and EUL data in this panel are identical to those shown in Figure 3 because the same experimental cohort was used.

**Fig. S8** EUL improves AIL via pathways involving gut microbiota-BAs-FXR signaling. EUL, flavonoids from *Eucommia ulmoides* Oliv. leaves; AILI, acetaminophen-induced acute liver injury. (A) Representative images of CK19 in liver tissues, scale bar=50 µm; Data are expressed as the median (min-max) and analyzed by student t test (n=6). (B-E) The levels of FGF15 in liver and serum; Data are expressed as the mean ± SEM and analyzed by ANOVA (n=5). All analyses of were based on a high dose of EUL (100 mg/kg). ^#^*P* < 0.05, ^##^*P* < 0.01, ^###^*P* < 0.001, compared with Ctrl group; ^*^*P* < 0.05, ^**^*P* < 0.01, ^***^*P* < 0.001, compared with APAP group. The APAP and EUL data in this panel are identical to those shown in Figure 7 because the same experimental cohort was used.

**Tab.S1** Identified compounds from water extract of EUL by HPLC-Q-TOF-MS/MS

| **NO.** | **Time(min)** | **m/z** | **Formula** | **Mode** | **MS^2^** | **Identification** | **ppm** | **Reference** |
| --- | --- | --- | --- | --- | --- | --- | --- | --- |
| 1 | 3.283 | 191.0557 | C_6_H_8_O_7_ | [M-H]^-^ | 173.0129，129.0472，111.0300，87.0484，85.2528 | Isocitric acid | 0.66 | (He et al., 2018; Jia et al., 2019) |
| 2 | 3.583 | 191.0614 | C_9_H_6_O_2_ | [M+COOH]^-^ | 129.04769，111.0302，87.0483 | Isocoumarin | -0.76 | mzCloud and ChemSpider |
| 3 | 5.101 | 191.0557 | C_7_ H_12_ O_6_ | [M-H]^-^ | 173.0456，87.0088 | D-(-)-Quinic acid | 0.66 | mzCloud and ChemSpider |
| 4 | 8.271 | 583.2659 | C_26_H_34_O_12_ | [M+COOH]^-^ | 375.0388，345.1344，327.1243，312.1004，297.0849，282.0889，191.0361，179.0238，151.0308 | Olivil 4'-O-glucoside | 4.80 | (He et al., 2018) |
| 5 | 10.123 | 757.1901 | C_32_H_38_O_21_ | [M-H]^-^ | 595.0688，462.0396，299.0201 | Quercetin glycoside 1 | -3.96 | (Huang et al., 2021; Tan et al., 2018) |
| 6 | 9.856 | 583.2665 | C_26_H_34_O_12_ | [M+COOH]^-^ | 537.2058，375.100, 360.1236，195.0503，165.0410，150.035 | Olivil 4''-O-glucoside | 4.20 | (He et al., 2018) |
| 7 | 10.173 | 353.0881 | C_16_H_18_O_9_ | [M-H]^-^ | 191.0366，179.0168，173.0278，135.0315 | Cryptochlorogenic acid | 3.86 | (He et al., 2018) |
| 8 | 10.273 | 431.1201 | C_18_ H_24_O_12_ | [M-H]^-^ | 269.0667，251.0563，165.0552，161.0456，143.0350 | Asperulosidic acid | 2.71 | mzCloud and ChemSpider |
| 9 | 10.724 | 625.1597 | C_27_H_30_O_17_ | [M-H]^-^ | 523.2048，195.0510，165.0420 | Quercetin 3-glucosyl- (1→6) -galactoside | -0.95 | mzCloud and ChemSpider |
| 10 | 11.375 | 459.1150 | C_18_H_22_O_11_ | [M+HCOO]^-^ | 191.0349，147.0445，119.04947 | Asperuloside | 0.92 | mzCloud and ChemSpider |
| 11 | 11.441 | 353.0882 | C_16_H_18_O_9_ | [M-H]^-^ | 191.0410，179.0206，135.0347 | Chlorogenic acid | 1.46 | (He et al., 2018; Huang et al., 2021; Jia et al., 2019; Li et al., 2022) |
| 12 | 11.608 | 353.0881 | C_16_H_18_O_9_ | [M-H]^-^ | 191.0558，179.0351，173.0456，161.02442，135.0451 | 4-O-caffeoylquinic acid | 1.42 | (Huang et al., 2021) |
| 13 | 12.209 | 353.0881 | C_16_H_18_O_9_ | [M-H]^-^ | 191.0558，179.0350，173.0456，161.02442，135.04515 | Neochlorogenic acid | 1.42 | (He et al., 2018; Huang et al., 2021; Jia et al., 2019) |
| 14 | 12.215 | 741.2053 | C_34_H_46_O_18_ | [M-H]^-^ | 417.1103,181.0247,151.0252 | Liriodendrin | 0.59 | (Huang et al., 2021; Jia et al., 2019) |
| 15 | 12.292 | 741.2048 | C_32_H_38_O_20_ | [M-H]^-^ | 417.1105,181.0250,151.0251 | Quercetin 3- (2G-xylosylrutinoside) | 0.11 | (Huang et al., 2021) |
| 16 | 12.459 | 177.0227 | [C_10_H_10_O_3_](https://pubchem.ncbi.nlm.nih.gov/#query=C10H10O3) | [M-H]^-^ | 164.0346，149.0098 | Coniferaldehyde | -2.57 | mzCloud and ChemSpider |
| 17 | 12.676 | 179.0344 | C_9_H_8_O_4_ | [M-H]^-^ | 135.0452，134.0733，107.0493，91.0346 | Caffeic acid | 3.10 | (He et al., 2018; Li et al., 2022) |
| 18 | 13.066 | 595.1289 | C_26_H_28_O_16_ | [M-H]^-^ | 475.0872，463.0906，200.0279，301.0322，271.02512，255.0330 | 2-(3,4-Dihydroxyphenyl)-5,7-dihydroxy-4-oxo-4H-chromen-3-yl 6-O-β-D-xylopyranosyl-β-D-glucopyranoside | 3.22 | mzCloud and ChemSpider |
| 19 | 13.517 | 595.1289 | C_26_H_28_O_16_ | [M-H]^-^ | 463.0432，301.0030，271.1501，255.0051，179.9836 | Quercetin 3-O-sambubioside | 3.22 | (He et al., 2018; Huang et al., 2021) |
| 20 | 13.801 | 609.1439 | C_27_H_30_O_16_ | [M-H]^-^ | 301.0040，300.6568，271.9987，255.0057，227.0111 | Rutin | 3.80 | (Huang et al., 2021; Li et al., 2022) |
| 21 | 14.585 | 579.1337 | C_28_H_36_O_13_ | [M-H]^-^ | 151.9962，181.0272 | (-)-Syringaresinol-4-O-beta-D-glucopyranoside | 3.53 | (He et al., 2018; Jia et al., 2019) |
| 22 | 14.952 | 463.0889 | C_21_H_20_O_12_ | [M-H]^-^ | 301.0042，300.7179，271.0003，255.0022，151.9930 | Isoquercitrin | 2.76 | (Huang et al., 2021) |
| 23 | 15.552 | 593.1487 | C_27_H_30_O_15_ | [M-H]^-^ | 285.0176，255.0096，227.0161 | Kaempferol-3-O-rutinoside | 4.22 | (Huang et al., 2021) |
| 24 | 16.613 | 161.0238 | C_9_H_6_O_3_ | [M-H]^-^ | 117.0337 | 4-Hydroxycoumarin | 2.73 | mzCloud and ChemSpider |
| 25 | 16.653 | 417.1561 | C_22_H_26_O_8_ | [M-H]^-^ | 387.0382，371.1823，181.0319，166.0116，137.0363 | Syringaresinol | 1.70 | (He et al., 2018) |
| 26 | 16.987 | 447.0926 | C_21_H_20_O_11_ | [M-H]^-^ | 285.0134，255.0084，227.0077 | Astragalin | 1.43 | (Tan et al., 2018; Yan et al., 2018) |
| 27 | 17.421 | 451.1037 | C_17_H_26_O_11_ | [M+COOH]^-^ | 387.0786，243.0523， 197.0399，191.0187，181.9058，179.0926，147.0439 | 8-O-Acetylharpagide | -0.09 | mzCloud and ChemSpider |
| 28 | 17.738 | 505.0980 | C_23_H_22_O_13_ | [M-H]^-^ | 301.0066，229.0310，179.9843，151.9928 | Quercetin-3-O-(6’’-O-acetyl)-β-D-glucopyranoside | 1.13 | mzCloud and ChemSpider |
| 29 | 17.848 | 451.1134 | C_21_H_24_O_11_ | [M-H]^-^ | 342.0410，209.6663，191.0150，180.8835 | Catechin 3'-O-beta-D-glucopyranoside | 1.32 | mzCloud and ChemSpider |
| 30 | 18.198 | 505.0996 | C_23_H_22_O_13_ | [M-H]^-^ | 301.0059，229.0312，179.9844，151.9926 | Quercetin 3- (6"-acetylgalactoside) | 3.91 | mzCloud and ChemSpider |
| 31 | 18.539 | 711.3949 | C_33_H_44_O_17_ | [M-H]^-^ | 549.1342，503.2818，357.0922，166.0101，151.9968 | medioresinol di-O-glucopyranoside | 1.42 | (He et al., 2018) |
| 32 | 19.416 | 187.1009 | C_9_H_16_O_4_ | [M-H]^-^ | 169.0875, 143.1059, 125.0977, 123.0794, 97.0680 | Eucommiol | -0.65 | (Huang et al., 2021; Shi et al., 2019) |
| 33 | 21.025 | 451.1042 | C_21_H_26_O_8_ | [M+COOH]^-^ | 342.0412，209.6660，191.0152，180.8836，165.9867，150.0338 | 1-(4-hydroxy-3-methoxyphenyl)-2-[4-(3-hydroxy-1-propen-1-yl)-2,6-dimethoxyphenoxy]-1,3-propanediol | -0.99 | (Huang et al., 2021; Shi et al., 2019) |
| 34 | 21.241 | 489.1047 | C_23_H_22_O_12_ | [M-H]^-^ | 285.0195，267.1486，255.0165，227.0204 | Kaempferol-3-O-(6''-O-acetyl)-β-D-glucopyranoside | -2.17 | (Huang et al., 2021) |
| 35 | 21.618 | 451.1139 | C_24_H_20_O_9_ | [M-H]^-^ | 342.0411，209.6659，191.0151，180.8836，165.9869 | Epigallocatechin 3-O-p-coumarate | 1.42 | mzCloud and ChemSpider |
| 36 | 21.835 | 489.1153 | C_23_H_22_O_12_ | [M-H]^-^ | 285.0194，267.1484，255.0168 | Quercetin 3- (2"-acetylrhamnoside) | 0.39 | mzCloud and ChemSpider |
| 37 | 27.047 | 301.0355 | C_15_H_10_O_7_ | [M-H]^-^ | 273.0405，255.0306，245.0455，151.0030，149.02441，121.0286， | Quercetin | 1.24 | (Huang et al., 2021; Li et al., 2022) |
| 38 | 34.505 | 285.0410 | C_15_H_10_O_6_ | [M-H]^-^ | 257.1026，159.5035，151.9036 | Kaempferol | 1.40 | (Huang et al., 2021; Li et al., 2022; Tan et al., 2018) |
| 39 | 35.132 | 285.0407 | C_15_H_10_O_6_ | [M-H]^-^ | 257.1027，159.5033，151.9041 | Luteolin | 1.40 | mzCloud and ChemSpider |
| 40 | 36.44 | 213.1512 | C_10_H_14_O_5_ | [M-H]^-^ | 183.1204，165.4481，151.3900，123.5044 | Guaiacyl glycerol | -6.99 | mzCloud and ChemSpider |

**Tab. S2** Primers for qPCR

| **Gene name** | **Forward sequence (5' to3')** | **Reverse sequence (5' to3')** |
| --- | --- | --- |
| FGFR4 | GGCTGTATTCCCCTCCATCG | CCAGTTGGTAACAATGCCATGT |
| CYP7A1 | CATCTCAAGCAAACACCATTCC | TCACTTCTTCAGAGGCTGGTTTC |
| Bsep | CAATGTTCAGTTCCTCCGTTCA | TTTGGTGTTGTCCCCSTSCTTG |
| OST-α | GTCTCAAGTGATGAACTGCC | TTGAGTGCTGAGTCCAGGTC |
| OST-β | GTATTTTCGTGCAGAAGATGCG | TTTCTGTTTGCCAGGATGCTC |
| GAPDH | CCAGCAAGAGCACAAGAGGA | GAGATTCAGTGTGGTGGGGG |

**Tab. S3** The VIP value of bile acid.

| **Bile acid name** | **VIP** |
| --- | --- |
| 7-ketoLCA | 1.87709 |
| DCA | 1.32252 |
| NorDCA | 1.25459 |
| TCA | 1.1494 |
| GCA | 1.05266 |
| CDCA | 1.04962 |
| ACA | 0.972933 |
| beta-MCA | 0.890081 |
| UDCA | 0.884937 |
| 6,7-diketoLCA | 0.858814 |
| T-beta-MCA | 0.766854 |
| CA | 0.734836 |
| UCA | 0.588175 |
| HDCA | 0.585103 |
| TDCA | 0.55848 |
| alpha-MCA | 0.484585 |

**References**

He, M., Jia, J., Li, J., Wu, B., Huang, W., Liu, M., Li, Y., Yang, S., Ouyang, H., Feng, Y., 2018. Application of characteristic ion filtering with ultra-high performance liquid chromatography quadrupole time of flight tandem mass spectrometry for rapid detection and identification of chemical profiling in Eucommia ulmoides Oliv. J Chromatogr A 1554, 81-91.

Huang, Q., Zhang, F., Liu, S., Jiang, Y., Ouyang, D., 2021. Systematic investigation of the pharmacological mechanism for renal protection by the leaves of Eucommia ulmoides Oliver using UPLC-Q-TOF/MS combined with network pharmacology analysis. Biomed Pharmacother 140, 111735.

Jia, J., Liu, M., Wen, Q., He, M., Ouyang, H., Chen, L., Li, J., Feng, Y., Zhong, G., Yang, S., 2019. Screening of anti-complement active ingredients from Eucommia ulmoides Oliv. branches and their metabolism in vivo based on UHPLC-Q-TOF/MS/MS. J Chromatogr B Analyt Technol Biomed Life Sci 1124, 26-36.

Li, X.Y., Fu, Y.J., Fu, Y.F., Wei, W., Xu, C., Yuan, X.H., Gu, C.B., 2022. Simultaneous quantification of fourteen characteristic active compounds in Eucommia ulmoides Oliver and its tea product by ultra-high performance liquid chromatography coupled with triple quadrupole mass spectrometry (UPLC-QqQ-MS/MS). Food Chem 389, 133106.

Shi, X.Q., Yue, S.J., Tang, Y.P., Chen, Y.Y., Zhou, G.S., Zhang, J., Zhu, Z.H., Liu, P., Duan, J.A., 2019. A network pharmacology approach to investigate the blood enriching mechanism of Danggui buxue Decoction. Journal of ethnopharmacology 235, 227-242.

Tan, Y., Zhao, H., Zou, L.S., Liu, X.H., CHAI, C., Wang, S.N., HUA, Y.J., 2018. Difference of chemical constituents in different medicinal parts of Eucommia ulmoides by LC-Triple TOF MS/MS. Journal of Chinese Mass Spectrometry Society 39, 101-111.

Yan, Y., Zhao, H., Chen, C., Zou, L., Liu, X., Chai, C., Wang, C., Shi, J., Chen, S., 2018. Comparison of Multiple Bioactive Constituents in Different Parts of Eucommia ulmoides Based on UFLC-QTRAP-MS/MS Combined with PCA. Molecules 23.
